# Multi-omics provides functional insights and underscores practical challenges in assessing the composition and performance of a nitrifying microbial consortium

**DOI:** 10.1128/aem.01984-25

**Published:** 2025-12-29

**Authors:** Derek D. N. Smith, Renuka M. Subasinghe, Caitlin Kehoe, Daniel S. Grégoire

**Affiliations:** 1 Environment and Climate Change Canada, Ottawa, Ontario, Canada; 2 Department of Chemistry, Carleton University 487910, Ottawa, Ontario, Canada; Universidad de los Andes, Bogotá, Colombia

**Keywords:** biotechnology, bioremediation, metagenomics, nitrogen metabolism, wastewater treatment, policy, microbial communities

## Abstract

Microbial consortia show promise for bioremediation of environmental pollution, but performance optimization and risk assessment remain challenging due to unculturable species and limitations of traditional biochemical and sequencing tools. This study demonstrates how a multi-omics approach can provide deeper insight into the performance and risks of using a model aerobic ammonia-oxidizing consortium under conditions representative of wastewater treatment. Long-read DNA sequencing recovered several high-quality genomes, revealing dominance by an unclassified *Nitrosospira* species with expected ammonia oxidation capabilities. Lower-abundance taxa with nitrogen cycling potential were also detected, though species-level identification was limited by poor taxonomic database representation. Multi-omics and nitrogen analyses showed shifts in community composition and nitrogen cycling activity when the consortium was grown along a redox gradient typical of wastewater. All cultures accumulated ammonia over 4 weeks, with only aerobic cultures reducing ammonia levels thereafter. The dominant *Nitrosospira* population declined in abundance and activity in aerobic cultures while shifting toward nitrogen reduction under anoxic conditions. This metabolic shift would not have been detected using amplicon sequencing alone. Multi-omics also supported risk assessment through detection of waterborne pathogens from the *Legionella* genus and other lineages harboring virulence genes resembling those from known pathogens. This study highlights the value of multi-omics for optimizing microbial consortia and assessing biosafety risks but also underscores challenges related to effective data analyses and the feasibility of risk assessment under realistic conditions. Addressing these challenges will be essential to support the broader adoption of multi-omics strategies by stakeholders working with microbial consortia across diverse environmental applications.

**IMPORTANCE:**

Microbial consortia are increasingly used to advance a sustainable bioeconomy. Optimizing consortia for environmental applications and ensuring regulatory compliance remains challenging, largely due to reliance on culturing microbes with unknown physiology. In this study, we apply cutting-edge sequencing to a consortium designed for ammonia removal from wastewater. Long-read DNA sequencing enabled complete genome recovery and revealed that populations integral to nitrogen cycling are poorly represented in taxonomic databases. By integrating multi-omics with biochemical assays, we uncovered how environmental conditions drive off-target nitrogen reactions and the potential risks of exposure to pathogens carrying virulence genes. Our findings underscore how whole-community approaches provide insights that are not obtainable with traditional amplicon sequencing and biochemical analysis methods. However, our study also provides recommendations on how hurdles related to data integration and environmental representation must be addressed to support stakeholders adopting such approaches in the context of commercializing microbial consortia.

## INTRODUCTION

Microbial communities consist of multiple microorganisms that rely on metabolic handoffs and symbiotic relationships to live in diverse habitats. These relationships can be leveraged toward societal benefits such as improving agricultural yields through the use of natural products, protecting human health with probiotics, and using microbial detoxification pathways to remove contaminants from aquatic and terrestrial ecosystems (1–6). Much of our understanding of using microbial pathways in those contexts stems from studies with well-characterized isolates and defined mixtures of strains. Although these studies are integral to providing mechanistic insights to support optimization efforts, cultivating stable consortia from environmental sources provides several advantages for stakeholders invested in scaling up sustainable biotechnology that can work in less controlled environments.

The cultivation of stable microbial consortia can preserve the complex metabolic interactions that occur *in situ*, which can be integral to maintaining the activity of pathways for environmental applications (e.g.*,* dechlorinating microbial consortia that rely on acetogens and methanogens to support reductive dechlorination) (7). Consortia cultivation allows for more environmentally relevant mechanistic studies by supporting stronger positive selection for key taxa with desired phenotypes under environmentally relevant conditions (8). Recreating these assemblages using mixtures of pure strains relies on all key members being cultivable, which poses a challenge because most microbes from environmental systems remain uncultivated (9, 10).

Consortia can be established through top-down approaches using environmental microbial communities as source material and continuously growing them under a pre-determined set of relevant conditions. Consortia can also be rationally defined using bottom-up approaches. Bottom-up approaches can rely on synthetic biology tools to introduce metabolic functions of interest, including through genetically tractable and modified strains and by controlling competition between different members within mixed communities (11). Both approaches present unique challenges that regulatory agencies must consider when assessing the risk of microbial products.

Synthetic biology can optimize desirable metabolic functions in scalable platforms but raises concerns about how genetically modified organisms can destabilize ecosystem services through competitive interactions and the introduction of mobile genetic elements (12, 13). Microbial consortia developed with top-down approaches raise similar concerns about their stability over time. Manufacturers must ensure a stable composition of consortia to preserve desired metabolic activity and limit off-target reactions across environmental conditions that deviate from optimized growth conditions (14, 15). Regulators must also consider the compositional stability of microbial consortia and the potential for off-target reactions that are potentially hazardous to evaluate the risks of consortia negatively affecting environmental and human health (12, 13, 16).

The main regulatory bodies tasked with assessing and approving commercial microbial remediation products in North America are Environment and Climate Change Canada (ECCC) and Health Canada (HC) for Canada, and the United States Environmental Protection Agency (US EPA). The Canadian Environmental Protection Act (CEPA 1999) (16) is the main statutory instrument through which non-pesticide microbial biotechnology products are regulated in Canada wherein microbial products are designated as notifiable substances containing living organisms (https://www.canada.ca/en/environment-climate-change/services/managing-pollution/evaluating-new-substances/biotechnology-living-organisms.html). Pure cultures of microorganisms and consortia are subject to regulatory approval and notification with ECCC/HC to be placed on the Domestic Substance List (DSL) with species-level identification of pure strains and community members. Consortia in this legal context only apply to natural assemblies of organisms, whereas synthetic communities require each organism to be classified as a substance subject to regulatory approval, if not on the DSL. In comparison to ECCC and HC, the US EPA focuses on regulating “new” organisms with genetic modifications that add novel functions in its regulatory framework via the Toxic Substances Control Act, with a large list of organisms (https://www.epa.gov/regulation-biotechnology-under-tsca-and-fifra/tsca-biotechnology-regulations).

Both processes rely on strain-level information obtained through cultivation work, which limits their ability to assess the risks tied to consortia that cannot be separated into cultivable isolates. Currently, Canada’s DSL has five consortia used for groundwater bioremediation that have completed the regulatory process, in comparison to 73 microbial strains added to the DSL via a new substance notification or included based on historical usage within Canada (17, 18) (Substances Search—Canada.ca Accessed 2024/11/08). Addressing this policy gap is critical to ensure regulators are equipped to assess the risks for microbial-based products for consumer and industrial applications (5, 19, 20). Additional analytical approaches can help support the assessment of a variety of products, including complex consortia and those suitable for widespread use in different environmental settings.

Cultivation-independent methods using short-read 16S rRNA amplicon sequencing can partially address these gaps, but these methods are limited because they lack the resolution required for strain-level characterization (21, 22). Emerging long-read metagenomic and metatranscriptomic sequencing are promising methods that can address the limitations of amplicon sequencing and short reads (23–26). Metagenomics can provide valuable insights into community metabolic potential and further validate taxonomy. These functional insights can help identify genes associated with pathogen virulence or undesirable metabolic reactions as well. These data can be leveraged alongside metatranscriptomics to identify which consortia members are metabolically active under a range of environmentally relevant conditions and consider which pathways are responsible for reactions that can have a negative impact on environmental chemistry.

Our objective is to demonstrate how multi-omics provides a functional lens that complements taxonomic analyses when assessing the risk of pathogen exposure and off-target reactions in microbial consortia. Additionally, we consider some of the practical challenges stakeholders may face when applying multi-omics strategies to optimize culture performance and carry out risk assessment under environmentally representative conditions. To meet these objectives, we worked with a model ammonia-oxidizing consortium being developed with a top-down approach for wastewater treatment plant (WWTP) settings. This consortium was chosen because WWTPs have served as models for community characterization using multi-omics, including multiple studies that use well-defined genetic targets to characterize ammonia (NH_3_) and more broadly, nitrogen cycling pathways (27, 28).

In this work, we assess community composition by combining long-read and short-read sequencing techniques. We monitor consortia performance driven by the key guilds required for NH_3_ removal (i.e.*,* nitrification) in WWTP: ammonia-oxidizing bacteria (AOB) and nitrite-oxidizing bacteria (NOB) that rely on metabolic handoffs to carry out nitrification (29). We use these tools to better understand how off-target reactions that negatively affect consortium performance can arise as communities respond to shifts in redox potential representative of wastewater. We use the same approaches to highlight the potential risks tied to pathogen exposure and virulence genes that must be considered when applying consortia in different settings. We conclude by providing recommendations that consider the limits of using multi-omics approaches in risk assessments to support building a reproducible framework that regulators can use in the future.

## RESULTS AND DISCUSSION

### Compositional and functional characterization of the starting consortium material

We identified 64 high-quality metagenome-assembled genomes (MAGs) from a single lot of the nitrifying consortium using a co-assembly approach that combined the long-reads from two DNA extraction kits used on fresh and frozen aliquots (Materials and Methods). All MAGs were classified to the Kingdom Bacteria with 13 unique phyla detected (sorted alphabetically): *Acidobacteriota, Actinomycetota, Armatimonadota, Bacteroidota, Bdellovibrionota, Chloroflexota, Deinococcota, Desulfobacterota_D, Eremiobacterota, Gemmatimonadota, Planctomycetota, Pseudomonadota,* and *Verrucomicrobiota*. A total of 42 unique families were identified, which included 16 unnamed families from the GTDB database (sheet #1 in Data S1). We also identified 46 unique genera, including 18 from unnamed genera (sheet #1 Data S1). Despite 60 of 64 MAGs recovered being 90% complete or higher, 10 MAGs were classified to unidentified genera, and 59 of the 64 MAGs could not be classified to the species level (sheet #1 in Data S1).

**Fig 1:**
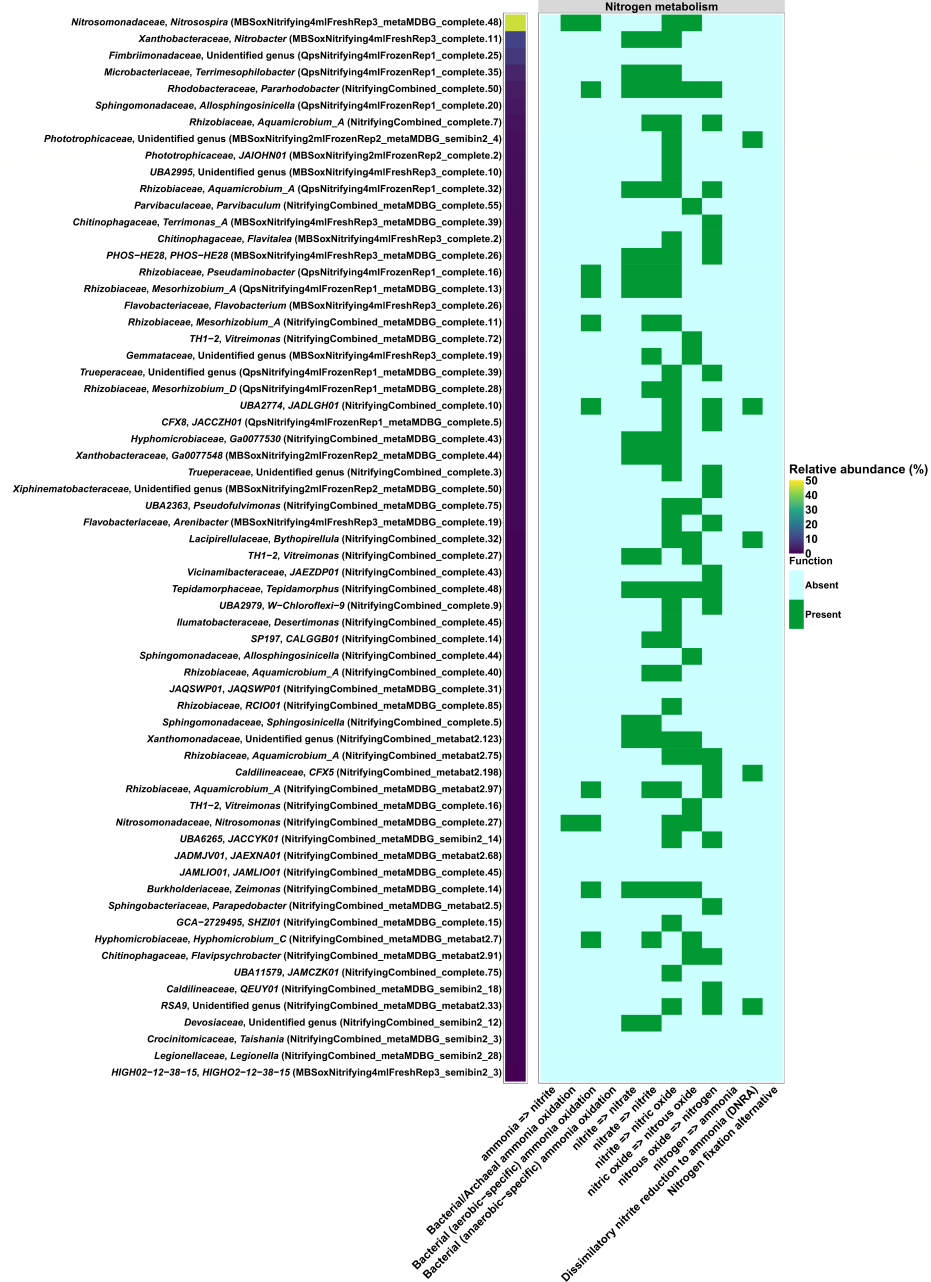
Relative abundance by sequence mapping and nitrogen metabolism for 64 MAGs built using PacBio long-read sequencing. Sequence abundance data were generated from the combined reads of multiple DNA extraction methods that were used to assemble MAGs.

To obtain a more representative view of the community structure, we combined the entire read sets in our sequence abundance analyses of the consortium starting material. MAG MBSoxNitrifying4mlFreshRep3_metaMDBG_complete.48 classified to the genus *Nitrosospira* (herein referred to as the dominant *Nitrosospira* population) maintained a high sequence abundance of 45.96%, whereas MBSoxNitrifying4mLFreshRep3_complete.11 classified to the *Nitrobacter* genus had an abundance of 9.73% (Fig. 1). Members of the *Nitrosospira* are known AOB that rely on handoffs to NOB, including members of the *Nitrobacter* genus to complete the oxidation of NH_3_ to nitrate (NO_3_^-^) (30, 31). These previous studies align with the detection of key genes for NH_3_ and nitrite (NO_2_^−^) oxidation in these populations (Fig. 1). Outside of the dominant *Nitrosospira* population, only MAG NitrifyingCombined_metaMDBG_complete.27 classified to the genus *Nitrosomonas* displayed the potential for NH_3_ oxidation (Fig. 1). This MAG occurred at a low abundance of 0.15%, suggesting it is not a major contributor to nitrification (sheet #1 in Data S1). No MAGs capable of the complete oxidation of NH_3_ (i.e.*,* comammox) or anaerobic NH_3_ oxidation (i.e*.,* anammox) were detected in the starting material (Fig. 1).

Beyond the dominant population of *Nitrosospira*, 11 MAGs had abundance values between 1% and 10% and the remaining 52 MAGs were <1% abundance (Fig. 1 and sheet #1 in Data S1). Several of the lower abundance MAGs carried genes for hydroxylamine oxidation to NO_2_^−^ and NO_2_^−^ oxidation to NO_3_^−^, suggesting they could assist in downstream steps of nitrification following NH_3_ oxidation by the dominant *Nitrosospira* population. Many of these MAGs also carried genes for denitrification, suggesting that oxidized nitrogen species could be lost from the culture through the production of N_2_ gas (Fig. 1).

None of the MAGs in the starting material carried genes for nitrogen fixation that could resupply NH_3_ in a setting in contact with N_2_ from the atmosphere or from denitrification (Fig. 1). Five MAGs with abundances <1.5% classified to unnamed genera carried the genes for dissimilatory NO_2_^−^ reduction to NH_3_, which could counteract nitrification activity (Fig. 1). Further analysis using DRAM identified five MAGs carrying the gene coding for the assimilatory NO_3_^−^ reductase catalytic subunit (i.e*.,* K00372) that could provide a source of NH_3_ (sheet #2 in Data S1). Although the potential for NH_3_ production through assimilatory and dissimilatory routes was detected, the low abundance of these populations in the starting material suggests they are not favored under conditions used to maintain the consortium.

Overall, the taxonomic and nitrogen cycling capacity of the consortium characterized using long-read sequencing aligns with its intended application in WWTPs. Genera containing AOB (e.g*.,* the dominant *Nitrosospira*) and NOB (e.g*., Nitrobacter*) occurred at high abundances, albeit not at a 1:1 ratio, suggesting the target guilds are positively selected for during cultivation. Deep long-read DNA sequencing also led to the recovery of lower abundance MAGs that potentially support downstream steps in nitrification or compete for nitrogen substrates via denitrification, dissimilatory NO_3_^−^ reduction, and assimilatory NO_3_^−^ reduction.

Despite many of these MAGs having between 99% and 100% completion, we were unable to classify most MAGs to the species level. We attribute the lack of species resolution to members of this consortium being poorly represented in GTDB despite a number of studies characterizing communities in WWTP settings using metagenomic techniques (27, 28, 32–34). Such database representation issues are likely more pronounced when using 16S rRNA, which lacks species resolution data. Improving the representation of microbial taxa from engineered systems where commercial consortia can be developed through top-down approaches will be important to leveraging multi-omics and amplicon sequencing strategies that provide compositional data that can be used by manufacturers and risk assessors to monitor consortia stability and safety.

### Cell growth and nitrogen cycling along a redox gradient

We conducted growth experiments for a total of 8 weeks in line with the medium refreshment schedule recommended for this consortium (Materials and Methods) to compare how the microbial community responsible for nitrogen cycling was affected by a redox gradient representative of WWTPs. All bottles that received live consortium material showed a threefold to fourfold increase in O.D. 600 nm over the course of the experiment up to ~0.4 (Fig. S1). No growth was observed in the sterile controls except for replicate #2 in the nitrate reduction treatment, which was likely contaminated around day 30 (Fig. S1). Aerobic and anaerobic cultures initially experienced a decrease in pH to 6.0, and only aerobic cultures saw a return to circumneutral pH values of 7.5 during the experiment (Fig. S1). These observations suggest that despite the manufacturer maintaining oxic conditions to select for AOB, the consortium in development can grow under anoxic conditions.

**Fig 2:**
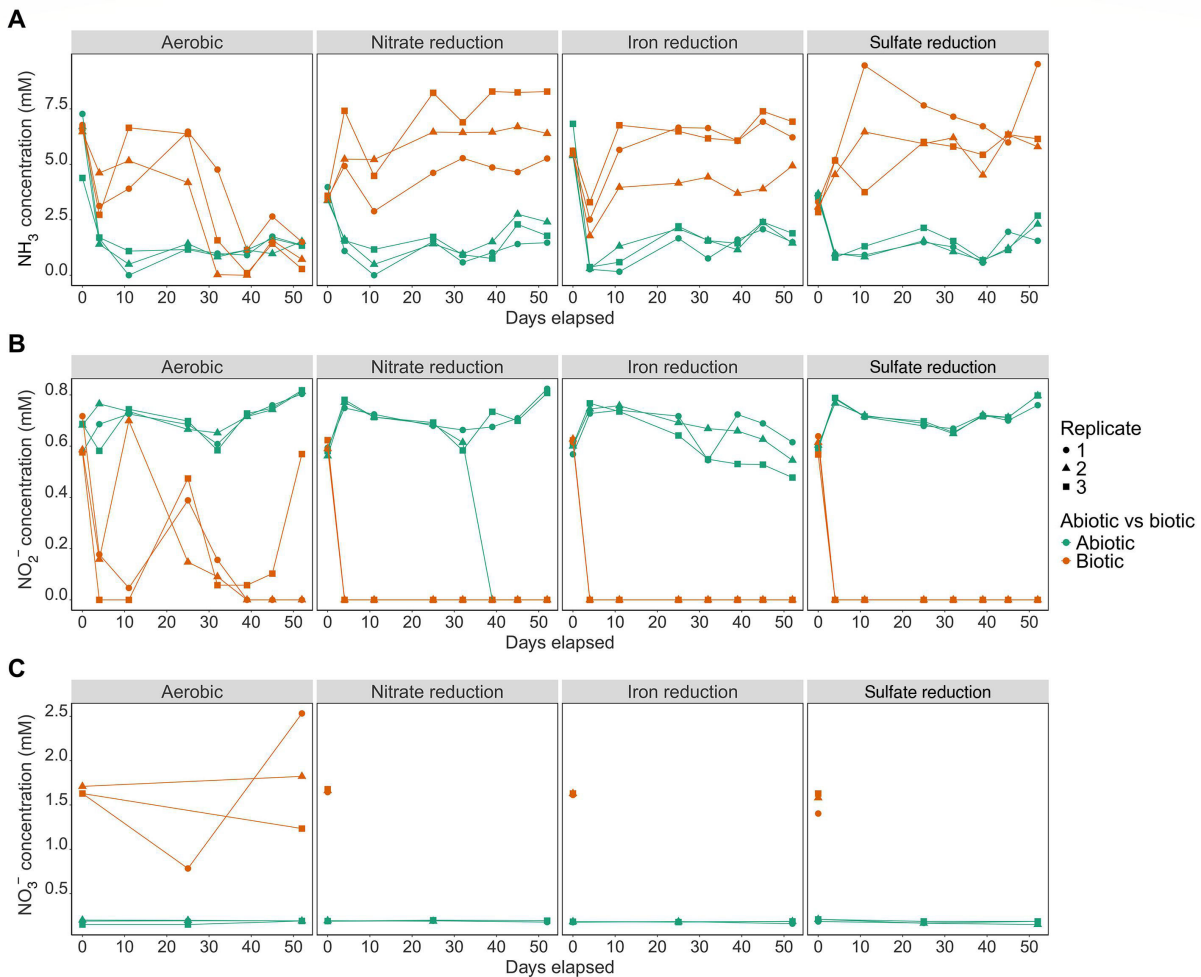
NH_3_ (**A**), NO_2_^−^(**B**), and NO_3_^−^ (**C**) concentrations for enrichment cultures inoculated with starting material from the nitrifying consortium in development and sterile controls grown along a redox gradient in synthetic pond water. NH_3_ and NO_2_^−^ were measured frequently during the incubation period using colorimetric methods, whereas NO_3_^−^ analyses could only be carried out for select samples tied to sequencing efforts. The aerobic cultures were purged daily with oxygen, and anaerobic cultures were maintained under a headspace of 97% N_2_/3% H_2_ and handled in an anaerobic glovebox. The nitrate reduction treatment was grown with the NO_3_^−^ already present in the medium acting as an electron acceptor, whereas the iron reduction and sulfate reduction treatments were amended with Fe^3+^ and SO_4_^2−^ as terminal electron acceptors, respectively. Abiotic vs biotic treatments are color-coded, and triplicates are denoted by different shapes.

Abiotic nitrogen cycling was not observed in sterile controls. Colorimetric NH_3_ measurements on day 0 were higher than the theoretical total nitrogen in the synthetic pond water (i.e*.,* 5.0–7.5 mM vs 3.11–4.11 mM) but stabilized to values in line with expectations afterward (i.e*.* 0–2.7 mM) (Fig. 2). NO_2_^−^ concentrations remained stable in abiotic controls and in line with the expected composition of the medium (ca. 0.47–0.82 mM) except for replicate #2 from the nitrate reduction treatment, which was contaminated (Fig. 2). NO_3_^−^ concentrations were stable in abiotic controls ranging from 0.19 to 0.21 mM, which were slightly lower than the theoretical 0.84 mM added to the medium (Fig. 2). We discuss sources of analytical variance for the nitrogen chemistry data in more detail in the *Nitrogen Chemical Analyses* section of the Supporting Results. Given that nitrogen measurements for all bottles were carried out identically and subject to the same limitations, it is suitable to compare nitrogen cycling over time through a phenotypic lens to support our microbiological analyses.

Live cultures differed considerably in the nitrogen cycling observed compared to abiotic controls. Aerobic cultures did not completely oxidize NH_3_ at the beginning of the experiment, and concentrations fluctuated over the ensuing weeks. Aerobic cultures initially decreased NH_3_ concentrations from ~7.0 mM, down to a range of 2.5–4.5 mM in the first 7 days; however, NH_3_ concentrations increased up to 5.0–7.5 mM between days 10 and 20 with considerable variance between replicates (Fig. 2). Additional decreases in NH_3_ concentrations below 2.5 mM occurred after 30 days in all aerobic cultures, suggesting a considerable lag period prior to net NH_3_ removal occurring (Fig. 2). The initial increases in NH_3_ in aerobic cultures were accompanied by fluctuations in NO_2_^−^ concentrations from 0.2 to 0.8 mM; however, the detection of 1.5−2.5 mM NO_3_^−^, the product of nitrification, did not occur until after day 30 when NH_3_ removal occurred more consistently (Fig. 2). In contrast to aerobic cultures, anaerobic cultures saw the total loss of NO_2_^−^ and NO_3_^−^ alongside plateaus in NH_3_ concentrations that were stable between 5.0 and 7.5 mM over the time frame of the experiment (Fig. 2). This trend was consistent regardless of the terminal electron acceptor supplied.

The pronounced lag in NH_3_ removal observed for aerobic cultures was unexpected. We attribute this lag to suboptimal growth conditions in the aerobic metabolic treatment. Our choice to supply oxygen periodically rather than continuously (Materials and Methods) potentially limited the oxygen required for NH_3_ oxidation and aerobic respiration. Although it is possible that some nitrification occurred over the first week of incubating the aerobic cultures, it is also possible that decreases in pH led to inhibition (Fig. S1). The manufacturer of the consortium in development indicated that aerobic nitrification is inhibited at pH <6.8 (personal communication), which is supported by other physiological studies on AOB where the lower pH favors the NH_4_^+^ ion vs the NH_3_ compatible with the AmoA protein responsible for oxidation (35). All aerobic cultures experienced pH decreases <6.8 within the first week of the experiment prior to pH returning to ~7–8, which may have prolonged the lag phase for nitrifiers (Fig. S1).

Regardless of the mechanisms that contributed to the lag, these observations highlight the potential for the consortium to shift toward NH_3_ production under oxygen limitations and time frames representative of WWTPs. Our own data show this suboptimal performance can be detected with chemical analyses; however, such tests offer limited insights into how a manufacturer could troubleshoot the biological processes affecting performance. Such troubleshooting is integral to manufacturers defining the recommended conditions where microbial consortia can be applied and risk assessors approving such usages to limit hazardous off-target reactions. We consider this perspective in the following section through a functional lens using metagenomics and metatranscriptomics to better understand what led to the phenotypic shifts observed.

### Shifts in taxonomic composition and nitrogen cycling in response to a redox gradient

Short-read DNA and RNA sequencing were used to characterize enrichment culture taxonomy and metabolic functions. Poor DNA yields due to low cell density and the costs of library preparation excluded the use of PacBio HiFi on DNA from our experimental data set despite our best efforts (Fig. S1). The use of short reads results in shorter contig assemblies with partial genes compared to long reads, which may influence mapping and resolution of closely related genes. Although we ran into the practical challenge of yield precluding the use of long-read sequencing, we attempted to counteract the potential bias tied to short reads by using a contig cutoff size of 500 bp, which strikes a good balance between including as many high-quality contigs as possible in assemblies and capturing complete or near-complete gene sequences to allow for multiple read tiling when mapping DNA and RNA via SqueezeMeta.

**Fig 3:**
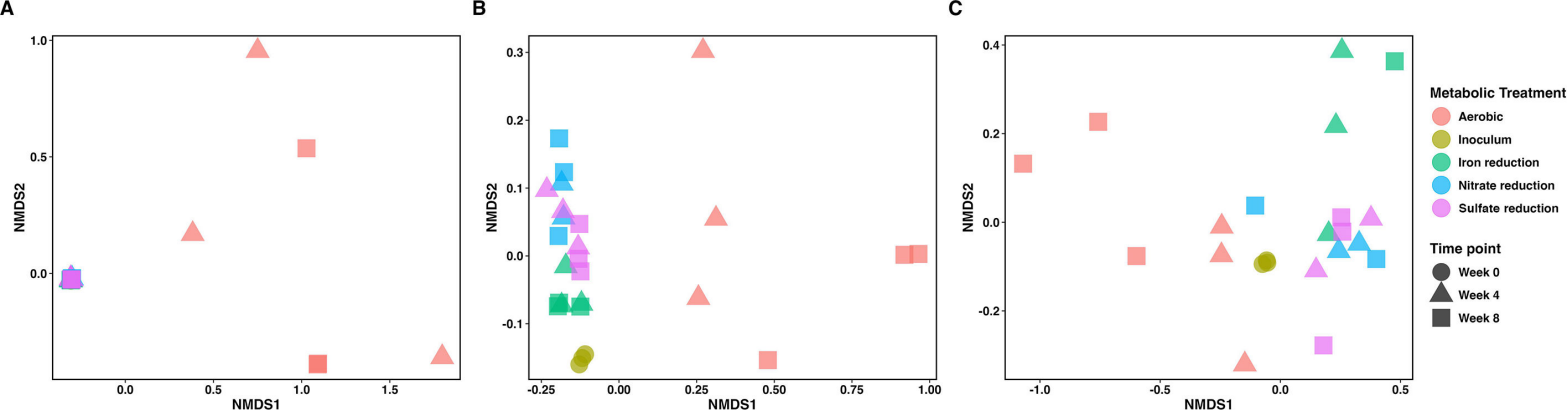
NMDS plots generated using the relative sequence abundance of MAGs (**A**), DNA read counts mapped to unique KEGG IDs in the coassembly (**B**), and RNA read counts mapped to unique KEGG IDs in the coassembly (**C**). Samples used for metagenomic and metatranscriptomic sequencing were taken from the nitrifying consortium inoculum and enrichment cultures grown along a redox gradient for 8 weeks. The Bray-Curtis dissimilarity index was used to generate a distance matrix required for NMDS analysis. The default command “metaMDS” from the “vegan” package was used to run 20 iterations of the NMDS ordination. The stress value for the MAG data in panel A was 0.0279, the stress value for DNA read-level data in panel B was 0.03, and the stress value for RNA read-level data was 0.075. Metabolic treatments have been color-coded, and the different time points from the incubation have been identified by different shapes.

Beta diversity analysis assessed at the MAG level using non-metric multidimensional scaling (NMDS) showed that anaerobic cultures maintained a similar composition as the starting inoculum (Fig. 3A). Aerobic cultures had variable MAG composition between replicates as the experiment progressed and diverged considerably compared to the inoculum and anaerobic cultures (Fig. 3A). When we omitted aerobic cultures from the MAG-level NMDS analysis, differences in community composition between anaerobic cultures and the inoculum were more apparent (Fig. S2). These observations show that relatively minor shifts in community composition occurred between the starting material and anaerobic cultures, and aerobic replicates experienced variable shifts in composition relative to the inoculum despite being grown in the presence of oxygen.

We repeated NMDS analysis using DNA and RNA reads mapped to unique KEGG IDs in the coassembly data to determine whether functional gene composition and transcription mirrored the MAG-level observations. NMDS analysis with DNA reads aligned with MAG-level observations. Aerobic cultures showed differences in gene composition between replicates and diverged considerably relative to the inoculum and anaerobic cultures in the ordination (Fig. 3B). Anaerobic cultures showed slight divergence from the inoculum in the DNA read-level data (Fig. 3B). These observations suggest that mapping DNA reads to the coassembly data discerned slight shifts in functional capacity that could not be detected in MAGs where considerable data are discarded in the process of generating high-quality genome bins.

NMDS analysis using RNA reads mapped to the coassembly differed from the MAG and DNA read data. Aerobic cultures from week 4 had a more similar RNA profile to the inoculum prior to diverging in composition at week 8, although variance between replicates was still observed (Fig. 3C). The similarity between the aerobic cultures and inoculum at week 4 is notable because it shows that the transcriptional profiles of both treatments were more similar during the period of NH_3_ removal despite shifts in MAG and gene composition (Fig. 2 and 3). RNA read-level analysis also showed differences between the anaerobic cultures that were not observed in the MAG or DNA read-level data. Samples from the nitrate and sulfate reducing treatments clustered close to the original inoculum in the ordination, but samples from the iron reduction treatment diverged relative to the other anaerobic treatments (Fig. 3C). These observations show that the redox gradient targeting different anaerobic guilds led to differences in gene expression despite MAG and DNA read-level composition being similar.

Given the focus of this study on using multi-omics to monitor nitrogen cycling performance, we examined the transcriptomic data related to nitrogen cycling in more detail. This was accomplished via mapping reads to genes present at the contig level. Short-read-level classification is significantly improved when using reference contigs and is the approach employed by SqueezeMeta (36, 37) (Materials and Methods). Long-read approaches available at the time of this study had significant technical and cost challenges that precluded their use in this case.

Nitrogen cycling genes accounted for ~1% of the total RNA reads mapped in the inoculum, and all samples from weeks 4 and 8 had < 0.25% of RNA mapping to nitrogen cycling genes (Fig. S3). These observations suggest there was likely a substrate limitation that led to a downturn in nitrogen cycling activity over the course of enrichment cultivation. We repeatedly observed maxima for RNA reads mapping to the *amoC* gene coding subunit C of the ammonia monooxygenase (i.e*.,* K10946) but not the *amoA* (i.e*.,* K10944) or *amoB* genes (i.e*.,* K10945) required for the functional enzyme complex (Fig. S3). There is evidence showing that nitrifiers from the *Nitrosospira* genus can upregulate *amoC* independently of *amoA* and *amoB* under NH_3_ starvation (38, 39). We suspect this occurred in our experiments based on NO_3_^−^ being carried over at the beginning of the incubation period, likely due to NH_3_ having been fully oxidized in the original inoculum material (Fig. 2). Notably, the increased RNA mapping to *amoC* was maintained in anaerobic cultures that were replete with NH_3_ throughout the experiment (Fig. S3). These observations suggest *amoC* undergoes distinct regulatory control related to redox potential, although this hypothesis requires formal testing. Relative differences in the % RNA mapped to nitrogen redox cycling machinery were discernible along the redox gradient used for cultivation. Aerobic replicates 2 and 3 showed low expression of *amo* and *hao* genes (i.e*.,* K10535), the latter coding the hydroxylamine dehydrogenase used for nitrification (Fig. S3). By contrast, aerobic culture replicate 1 had more RNA mapping to nitrous oxide (i.e*.,* K00376) and nitrite reductases (i.e*.,* K00368) (Fig. S3). Aerobic cultures also showed low RNA mapping to nitric oxide reductase subunit B (i.e*.,* K04561) (Fig. S3). The comparably low % of RNA mapping to nitrification and denitrification machinery supports that periodic oxygen supplies limited aerobic nitrification to the point that low activity of anaerobic denitrification machinery was maintained in this experimental treatment.

RNA reads did not map to the *amoA* or *amoB* genes in the anaerobic cultures but did map to genes coding denitrification machinery, which aligns with the requirement for oxygen to support nitrification (Fig. S3). The proportion of RNA reads mapped to nitrogen cycling in the iron reduction treatment was similar to the nitrate and sulfate reduction treatment, suggesting that the divergence in the transcriptional profiles noted previously is occurring in genes other than those involved in nitrogen cycling (Fig. S3). Most anaerobic cultures had higher proportions of RNA reads mapping to nitrous oxide and nitrite reductases and small proportions of RNA mapping to nitrite reductase (cytochrome c-552) (i.e*.,* K03385) (Fig. S3). RNA reads mapping to the nitrite reductase (cytochrome c-552) coded by the *nrfA* gene is notable, because this gene is considered a marker of dissimilatory NO_2_^−^ reduction to NH_3_ that could be contributing to NH_3_ production (40, 41). These observations suggest that anaerobic cultures shifted their metabolism to anaerobic reduction of oxidized nitrogen species in line with the nitrogen chemistry data, despite little detectable change occurring in the community structure relative to the starting inoculum.

The unexpected compositional differences observed in aerobic vs anaerobic cultures and contrasting nitrogen cycling phenotypes prompted us to examine the metabolic contributions of nitrogen cycling guilds in more detail. Although we used % RNA mapping data to support our interpretations, we acknowledge that a large proportion of RNA did not map to the MAGs (Fig. S4). Further examination showed that this discrepancy was due to a large proportion of reads mapping to contigs that were not captured in genome bins. This may be due to a combination of challenges in resolving full MAGs of organisms at sufficient depth in subsets of our experimental samples using short reads for metagenomic binning that long reads could have addressed if it were technically feasible in this instance. We consider how RNA data align with sequence abundance and functional gene presence with this caveat in mind in this section.

**Fig 4:**
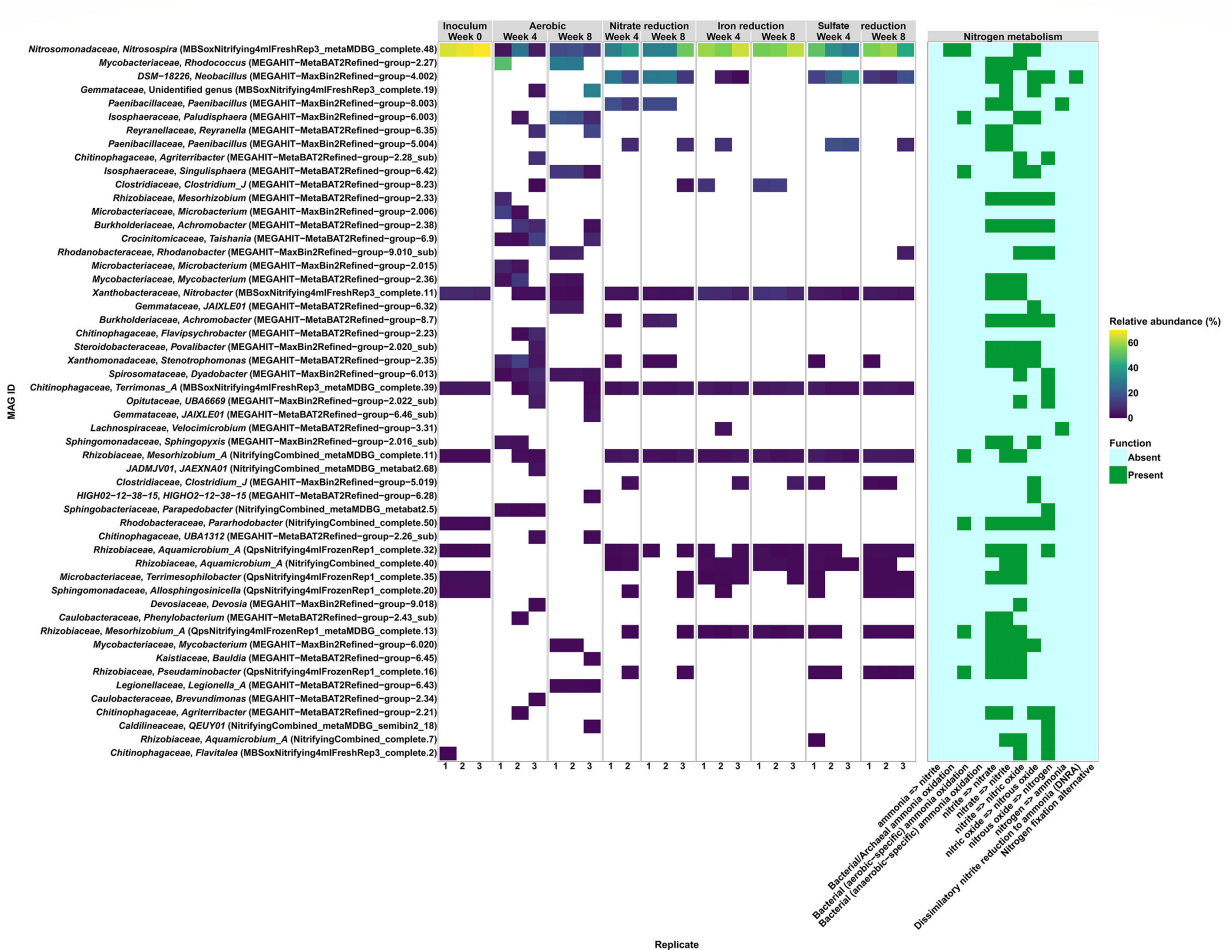
Sequence abundance for MAGs ≥ to 1% and nitrogen metabolism for enrichment cultures established with the nitrifying consortium in development as starting material and grown along a redox gradient for 8 weeks. Enrichment culture samples were exclusively sequenced using Illumina shotgun metagenomic sequencing. All MAGs assembled from the starting material and enrichment culture time series were combined into a final dereplicated series of MAGs to assess relative abundance.

Aerobic cultures saw a decrease in the abundance and % of RNA reads mapped to the dominant *Nitrosospira* (i.e*.,* MAGMBSoxNitrifying4mlFreshRep3_metaMDBG_complete.48). This MAG had abundances of ~65% to 70% at Week 0 and accounted for 36%–42% of the total RNA reads mapped in the starting material (Fig. 4; Fig. S4). The abundance, % total RNA mapped, and % RNA mapped classified to nitrogen cycling genes in the *Nitrosospira* genus decreased as the incubation progressed (i.e*.,* 3%–26% abundance, <2.5% for total RNA, <0.2% RNA for nitrogen cycling genes) (Fig. 4; Fig. S4 and S5). These decreases occurred alongside lower abundance of MAG MBSoxNitrifying4mlFreshRep3_complete.11 from the NOB genus *Nitrobacter* (i.e., ~10% to <2%) and no RNA mapping to *Nitrobacter* nitrogen cycling genes (Fig. 4; Fig. S4 and S5). These results support that nitrifying guilds decreased activity in the aerobic treatment but maintained low nitrification after a lag period.

Aerobic cultures also experienced an increase in the sequence abundance of MAGs with the capacity to contribute to nitrogen cycling that were not detected in the inoculum. MAG MEGAHIT−MetaBAT2Refined−group−2.27, which was classified to *Rhodococcus* sp. 00434560, carried genes for NO_2_^–^ oxidation and partial denitrification (Fig. 4). This *Rhodococcus* had a sequence abundance ranging from 27.78% to 48.82% and accounted for 0.1% to 30% of the total RNA mapping to a given MAG, supporting that they were metabolically active in these cultures (Fig. 4; Fig. S4). Whether this population directly contributed to nitrogen redox cycling is difficult to discern, given that the % RNA mapped to nitrogen cycling genes from this genus was low, like all other genera noted in these analyses (Fig. S5). MAG MEGAHIT−MaxBin2Refined−group−6.003, which was classified to an unidentified species in the *Paludisphaera* genus, also carried genes for hydroxylamine oxidation and partial denitrification (Fig. 4). This population maintained an abundance between 7.59% and 18.82 % and accounted for 1%–6 % of the total RNA mapping to MAGs (Fig. 4; Fig. S4). No RNA mapping to nitrogen cycling genes from this genus could be detected, suggesting they were not a major contributor to nitrogen cycling in these cultures, although taxonomic classification of functional genes rarely went deeper than family level in most cases (Fig. S5).

Major shifts in the relative abundance of the dominant AOB population of *Nitrosospira* were not apparent in the anaerobic cultures, which likely contributed to the compositional similarity noted in MAG-level NMDS analysis (Fig. 3A). Most anaerobic cultures maintained *Nitrosospira* populations at sequence abundances between 29.10% and 64.53%, which is comparable to the 65%–70% observed at week 0 (Fig. 4). The NOB *Nitrobacter* population had abundances ranging from 2.18% to 9.61%, comparable to the starting material (Fig. 4).

Despite little change observed at the MAG level, there was considerable variance in the % RNA mapped to MAGs from nitrifying guilds and MAGs that precluded detection in the inoculum. The most notable difference was the % of RNA mapping to MAG MEGAHIT-MaxBin2Refined-group-4.002, which ranged from 1% to 78% (Fig. S4). This MAG represents an unidentified species in the *Neobacillus* genus that ranged in abundance from 0.19% to 35 % and carried genes for partial denitrification and dissimilatory NO_2_^−^ reduction to NH_3_ (Fig. 4). Anaerobic cultures saw variable % RNA mapping to this MAG and nitrogen cycling genes alongside the key *Nitrosospira* population (i.e*.,* 0.5%–30%), suggesting they were competing for nitrogen substrates (Fig. S4 and S5). Similarly, MAGs classified to the *Paenibacillus* genus with the capacity for partial denitrification and nitrogen fixation (i.e*.,* MEGAHIT-MaxBin2Refined-group-5.004 and MEGAHIT-MaxBin2Refined-group-8.003) ranged in sequence abundance from 0.9% to 23% despite not being detected in the inoculum (Fig. 4). We highlight these MAGs because they accounted for 0.5% to 23% of the RNA mapped to bins in some cases, demonstrating they were metabolically active in the community (Fig. S4). Whether the detection of nitrogen fixation potential in these populations contributed to NH_3_ detected in the anaerobic cultures is unclear, given the limitations noted for taxonomic assignment of nitrogen cycling gene transcripts (Fig. S5).

Although specific populations were detected in anaerobic cultures that could contribute to NH_3_ through assimilatory, dissimilatory, and nitrogen fixation pathways, there was no evidence of these pathways in aerobic cultures that saw comparable increases in NH_3_ concentrations for the first 4 weeks of the incubation (Fig. 4). Instead, we suspect that aerobic cultures were able to access undefined sources of NH_3_ contained in the yeast extract that was supplied to mimic complex nutrients typically found in WWTPs (42). When triaging metatranscriptomics results to the top 25 values obtained for RNA read counts associated with KEGG IDs for amino acid metabolic pathways, we observed increased transcription for genes coding for glutamate dehydrogenase and glutamine synthetase that can catalyze deaminating reactions to produce NH_3_ in aerobic cultures (Fig. S6). Anaerobic cultures also increased transcription of genes coding enzymes capable of deamination, including glutamine synthetase and glutamate synthase that could contribute to NH_3_ alongside the nitrogen cycling pathways noted previously (Fig. S6).

The functional analyses of the enrichment cultures reinforce that the key *Nitrosospira* population required for nitrification can shift their metabolism in response to redox potential. This shift can occur alongside the growth of new populations that preclude detection in the starting material, with the capacity for undesirable nitrogen transformations that affect consortium performance. WWTPs routinely experience oxygen limitations and are known to support obligately anaerobic guilds (43). We highlight this environmental context to underscore how using a multifaceted approach that considers compositional stability across replicated cultures over time alongside gene presence and absence, and transcription, provides manufacturers with comprehensive information that can be used to optimize consortium performance.

Although regulators are concerned about pathogenic organisms, the potential for off-target reactions that pose environmental hazards is also an important consideration. Indeed, if we had taken a traditional approach and solely characterized the consortium starting material via high-level taxonomic analyses, we would not have been able to detect these shifts in composition, functional capacity, and nitrogen cycling activity. It is only once we considered the genes present and their contributions to metabolic activity that we discerned how cultures with similar composition supported contrasting phenotypes relevant to the application of this consortium. We turn this functional lens towards pathogen and virulence gene detection in the next section to emphasize the utility of such approaches for supporting regulatory priorities.

### Pathogen detection and virulence assessment

**Fig 5:**
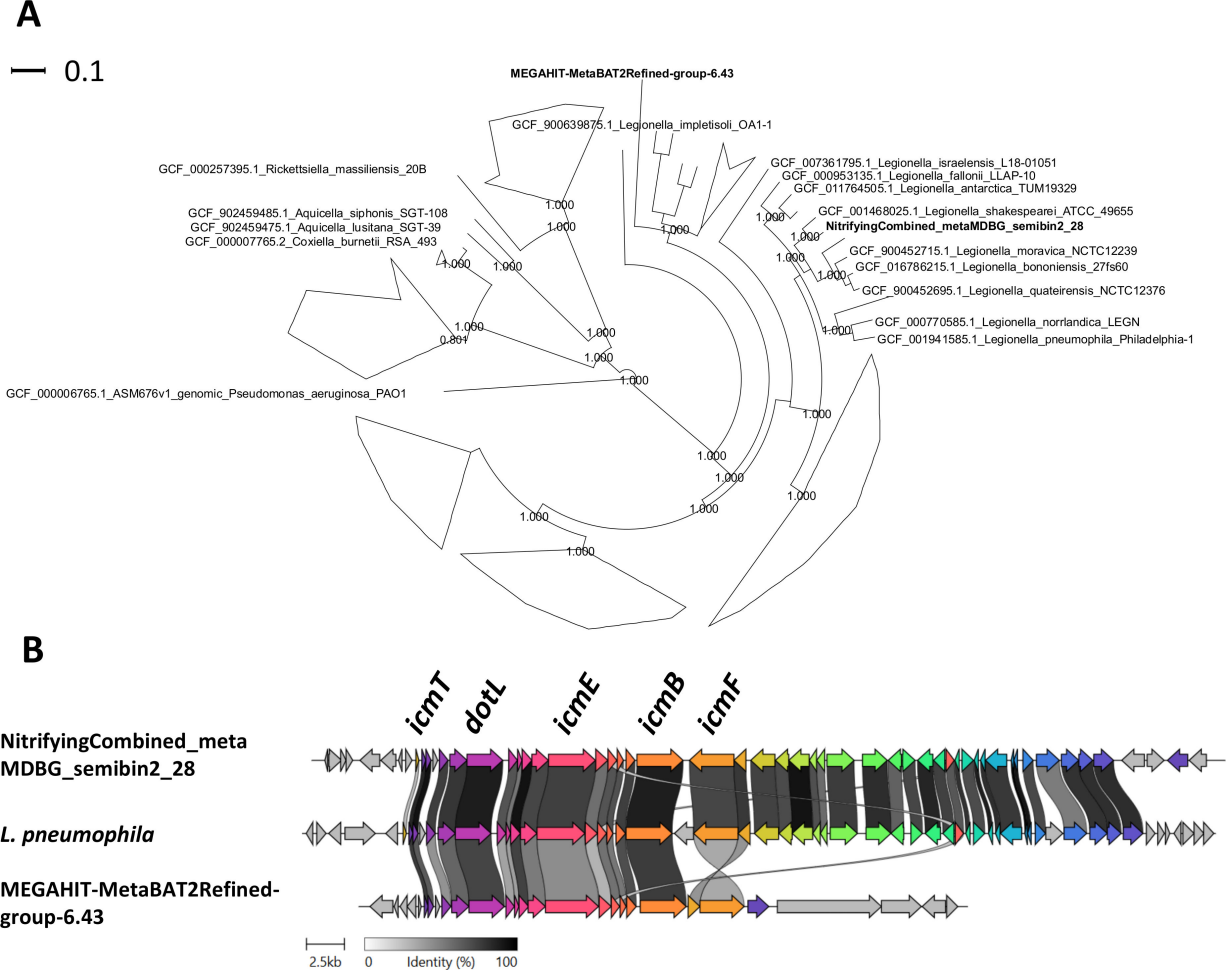
Phylogenetics and comparative genomics of *Legionella* MAGs recovered from consortium inoculum and experimental treatment. (**A**) The phylogenetic tree was generated from single-copy genes identified via GtoTree using the Gammaproteobacteria HMM profile (up to 172 genes) and was rooted using the genome of *Pseudomonas aeruginosa* PAO1. Clades were collapsed, and nodes were hidden for clarity and to highlight relationships of reference genomes in the order *Legionellales*. (**B**) Clinker was used to compare genomic regions containing a Type IV secretion system extracted from contig 17 of NitrifyingCombined_metaMDBG_semibin2_28 and contig 7 of MEGAHIT-MetaBAT2Refined-group-6.43 and the chromosome of *L. pneumophila* (GCA_001941585.1, BioSample: SAMN03988484).

The ambiguity of taxonomic identification of whole MAGs from environmentally derived consortia is important to consider in the context of pathogen detection. Our use of long-read sequencing in this study generated genomes with high levels of completeness. This allowed for the detection of a MAG classified to an unnamed species in the *Legionella* genus (i.e., NitrifyingCombined_metaMDBG_semibin2_28, 93.7% complete, sheet #4, Data S1), which includes known pathogens associated with WWTPs (44, 45). An additional MAG from the *Legionella* genus (i.e*.,* MEGAHIT-MetaBAT2Refined-group-6.43, 99.7% complete, sheet #4 Data S1) was also detected after prolonged aerobic enrichment at week 8 with short reads (Fig. 4). The *Legionella* MAGs differed in size and %GC content, wherein MAG MEGAHIT-MetaBAT2Refined-group-6.43 was 1,881,538 bp with 32.1% GC in comparison to MAG NitrifyingCombined_metaMDBG_semibin2_28 which was 3,340,288 bp and 38.9% GC. MAG NitrifyingCombined_metaMDBG_semibin2_28 was most closely related to *Legionella shakespearei* based on a phylogenetic tree generated from multiple single copy genes (Fig. 5A). MEGAHIT-MetaBAT2Refined-group-6.43 formed a monophyletic group and shared a later common ancestor with *Legionella pneumophila* when compared to MAG NitrifyingCombined_metaMDBG_semibin2_28 (Fig. 5A). Both MAGs contained elements of the Type IV Dot/Icm secretion system essential for pathogenesis and broadly distributed across *Legionella* species (46) (Fig. 5B) with lower identity for genes identified in MEGAHIT-MetaBAT2Refined-group-6.43 relative to *L. pneumophila* (sheet # 5, Data S1).

Although we highlight the example above for *Legionella* because of its history as a waterborne pathogen and detection in both starting consortium material and enrichment cultures, additional MAGs above or at 1% sequence abundance classified to other lineages containing pathogens were recovered from enrichment cultures. These MAGs included MAG MEGAHIT-MetaBAT2Refined-group-8.7 (100% complete, Fig. 4, sheet #4 Data S1), which was taxonomically classified as *Achromobacter denitrificans* (Fig. S7). *A. denitrificans* is a risk group 2 human pathogen according to the Public Health Agency of Canada, and this MAG contained a putative antibiotic resistance gene with 80% nucleotide identity to the chromosomal *AXC*-2 from *Achromobacter ruhlandii* (CARD ARO:3,006,898 [47]), homologs of this gene having potential for hydrolysis of carbapenems (48) (sheet #6, Data S1). In non-pathogens, the presence of antibiotic resistance genes could be an important consideration for risk assessment, particularly if there is clear contextual evidence of transmissibility.

Several MAGs were also recovered and classified to the family *Mycobacteriaceae*. MAG MEGAHIT-MaxBin2Refined-group-6.020 (94% complete, sheet #4, Data S1) was classified as *Mycolicibacter longobardus* (formerly *Mycobacterium longobardum*), MEGAHIT-MetaBAT2Refined-group-2.36 (100% complete, sheet #4, Data S1) was classified as *Mycolicibacterium phocaicum* (formerly *Mycobacterium phocaicum*), and MEGAHIT-MetaBAT2Refined-group-2.27 (100% complete, sheet #4, Data S1) was classified as *Rhodococcus* sp004345605 (Fig. S8). This family includes many pathogenic species, with *M. longobardus* and *M. phocaicum* having case reports of human infection (49–51). Multiple putative virulence factors were identified in the *M. longobardus* MAG, some of which had greater than 80% nucleic acid identity to virulence factors in *Mycobacterium tuberculosis* (sheet #5, Data S1). These data further highlight the value of compositional analyses under representative growth conditions coupled to shotgun metagenomic approaches to identify organisms with potential functions of concern.

### Conclusions

In this study, we successfully applied long-read DNA and short-read DNA and RNA sequencing to characterize a consortium in development for NH_3_ removal from wastewater. Our results showcase the potential for multi-omics to address industry and regulatory stakeholder needs. We demonstrated that long-read sequencing enabled the recovery of nearly complete genomes from microbes whose functions aligned with the intended use of this consortium for NH_3_ removal. Long-read sequencing ran into practical limitations with yield and costs within our experimental setup, and we used short-read approaches to circumvent these challenges. Whole-community techniques using short reads revealed that taxa not initially detected in the starting material could become abundant and contribute to changes in nitrogen cycling activity that can affect consortium performance while still detecting major constituents identified in the long-read sequenced inoculum.

From an industry perspective, maintaining consortium performance over a wide range of environmental applications is critical to the widespread and safe use of microbial products. Although performance can be monitored with straightforward chemical analyses in many cases, applying a functional lens provides insights into the biological processes contributing to off-target and/or potentially hazardous reactions. In the case of human health, whole-community approaches can help detect putative pathogens based on virulence functions. These approaches can address limitations in detecting new or emerging pathogens that preclude cultivation with standard tests and are an alternative to sequencing techniques that may provide insufficient taxonomic resolution.

Our work also revealed the practical challenges of applying multi-omics to characterize microbial consortia. Long-read sequencing provides substantial benefits in microbial metagenomics for identifying taxa and functional genes, yet practical challenges required the usage of short reads to thoroughly characterize both our consortium inoculum and experimental samples. Despite using long-read and short-read metagenomic sequencing for taxonomic classification, many taxa in this consortium lacked species-level representation in commonly used databases. These challenges will persist for sequencing-based approaches as long as certain habitats remain undersampled and cultivated representatives remain challenging to obtain. Although RNA sequencing provided functional insights relevant to consortium performance, depleting rRNA and connecting these data to DNA sequencing results required considerable expertise, time, and computational resources. Analyzing these data were made more challenging by the variance observed in nitrogen cycling, community composition, and transcriptional responses in enrichment cultures started from the same inoculum source. Some of this variance could stem from the capacity to confidently resolve MAGs and longer contigs bearing functional genes using short-read sequencing. Although this may be resolved with long-read sequencing, our study underscores that this is not always possible for practical reasons tied to culture growth and feasibility.

The scenarios outlined above show that it is unrealistic for stakeholders to try to capture how consortia composition and function may shift along an indeterminate combination of environmental variables. There is a clear need for a multi-omics framework that can be reproduced across microbial products with different metabolic functions. Our research supports the utility of a framework that includes replicated small-scale applications of microbial consortia under a set of representative conditions to profile community composition and fates. However, the complexity of multi-omics analyses may require triage strategies that stakeholders can use to identify cases when in-depth whole-community analyses are warranted or when simple chemical or standard microbiological tests will do. Standardizing the use of user-friendly bioinformatics pipelines to apply to complex data sets enabling analysis of whole-community data will be critical to acquiring salient information to advance decision-making. These approaches could be co-developed with industry stakeholders to ensure they consider relevant analytical endpoints across the broad range of applications for microbial consortia. Our work makes a strong case for incorporating whole-community genomics approaches as a foundation of reproducible frameworks through which microbial consortia are assessed in the future.

## MATERIALS AND METHODS

### Initial consortium maintenance

This work was carried out in collaboration with an industry partner that has requested anonymity. The partner kindly provided a consortium that was in development for removing NH_3_ from wastewater. This culture was maintained in a proprietary defined medium under constant aeration. The medium was refreshed every 6–8 weeks, which informed the timeframes applied in our growth experiments. All other information related to culture maintenance is proprietary.

### Long-read PacBio sequencing on the starting consortium material

A single bottle containing ~1 L of culture of the nitrifying consortium in development was shipped at room temperature and stored at 4°C after receipt. Aliquots were frozen in sterile 15 mL conical tubes and stored at −80°C to preserve the culture profile, with 4 mL of thawed sample being used for extraction or 2 mL of fresh culture. Cell pellets were extracted using the PowerSoil Pro kit (Qiagen) and the Sox DNA Extraction kit (Metagenom Bio Life Science). 4 µL of RNase A (100 mg/mL) was added after each kit’s bead beating step, and tubes were incubated for 5 min at room temperature to reduce RNA that may interfere with PacBio sequencing. Samples were purified and concentrated with the Genomic DNA Clean & Concentrator-10 Kit (Zymo Research). PacBio Sequel II sequencing was performed at Integrated Microbiome Research (Dalhousie University, Halifax, NS) using the SMRTbell Prep Kit 3.0 for shotgun metagenomics. The samples were already sheared sufficiently for library preparation from the extractions, and 1.82 million (22.6 Gbp) total reads were generated from three samples from the two extraction kits.

### Enrichment experiment setup

A fresh sample from the same lot of the nitrifying consortium in development previously sequenced by PacBio was used to inoculate enrichment cultures that were grown in a synthetic pond water recipe provided by the consortium manufacturer. Synthetic pond water was prepared by dissolving the ingredients listed in Table 1 in 1 L of ultra-filtered water (i.e*.,* Milli-Q) prior to adjusting pH to 7 with 0.5 mL of 1 M NaOH and autoclaving at 121°C for 30 min.

**TABLE 1:** Synthetic pond water recipe

Medium component	Reagent	Concentration (g L^−1^)	Concentration (mM)
Basal medium	KNO_3_	0.085	0.84
	NaNO_2_	0.06	0.87
	NH_4_Cl	0.075	1.4
	H_3_PO_4_	0.038	0.39
	Yeast extract	1	NA^*a*^
	Glucose	1	5.55
Fe^III^ stock (1 L) 100× (10 mL in 1 L medium)	Fe^III^-ammonium citrate^d^	0.262	1
SO_4_^2−^ stock (1 L) 100× (10 mL in 1 L medium)	Na_2_SO_4_	0.142	1

^*a*^ No concentration for yeast extract is provided (NA) in mM due to its unknown molar mass.

We prepared cultures and abiotic controls for the different metabolic rates in triplicate by filling 90 or 100 mL of medium, respectively, into 150 mL serum bottles sealed with butyl rubber stoppers that were crimped shut. Aerobic cultures were filled with basal medium stored on the bench and purged daily on working days, Monday to Friday, with a sterile supply of atmospheric air that passed through an autoclaved 0.22 µm pore-size PTFE filter. We chose to grow aerobic cultures in this way rather than in containers open to the atmosphere that were shaken to ensure consistent containers and sampling equipment were used for all cultures in this experiment. The medium used for anaerobic treatments was bubbled under a sterile flow of N_2_ for 30 min prior to being moved to a Coy anaerobic glovebox that maintained an atmosphere of 97% N_2_/3% H_2_ for aliquoting into serum bottles conditioned under anoxia for 48 h. The anaerobic metabolic treatment for nitrate (NO_3_^−^) reduction was not amended with any other reagents because NO_3_^−^ was already present in the medium (see Table 1). Metabolic treatments for iron reduction and sulfate reduction were amended to a final concentration of 1 mM of Fe^3+^ or SO_4_^2−^ from 100-fold stocks that were filter-sterilized and bubbled under N_2_ prior to use (see Table 1).

Sterile abiotic controls did not receive any material for inoculum. Biotic samples were inoculated at 10% (vol/vol) using biomass from a freshly received batch of the consortium using sterile 10 mL syringes. Inoculum material for aerobic cultures was handled on the bench, and the same material was subsequently bubbled under sterile N_2_ prior to inoculating anaerobic cultures to minimize oxygen intrusion. A 5 mL subsample was withdrawn from the starting material in addition to abiotic and biotic bottles prior to being filtered through a 0.22 µm pore size PES filter, measured for pH and redox potential, and stored at −20°C until nitrogen chemistry analysis. 2 mL aliquots were also obtained in triplicate for RNA extraction. These samples were placed in sterile conical tubes and topped up with 10 mL of DNA/RNA shield (Zymo Research) and stored at −20°C until processing. An additional 2 mL aliquots were withdrawn in triplicate from the starting material for DNA acid extractions. These samples were centrifuged at a speed of 10,000 × *g* for 8 min prior to decanting the supernatant and storing pellets at −80°C until processing.

Abiotic and biotic bottles had 5 mL withdrawn weekly for nitrogen chemistry, pH, and ORP measurements as outlined above. Additional 0.2 mL aliquots were also retrieved to measure cell growth at an OD of 600 nm in a 96-well plate using a plate reader with a monochromator. Subsamples for nucleic acid extraction were withdrawn bi-weekly and stored as noted above.

### Nitrogen chemistry analyses

NO_2_^−^ and NH_3_ were measured using dedicated colorimetric methods adapted from the literature. A select number of subsamples were sent to an independent certified lab (i.e*.,* Bureau Veritas, ISO 17025) to carry out NO_2_^−^ and NO_3_^−^ analyses for select archived time points that were representative of the beginning, middle, and end of the experiment using the SM 23 4500-NO_3_I/NO_2_B protocol. NO_2_^−^ analyses were conducted using a modified Griess-reagent-based method (Invitrogen, G7921). The Griess reagent was prepared by mixing equal parts of N-(1-naphthyl)ethylenediamine and sulfanilic acid. Samples were thawed daily and diluted 10-fold in a modified synthetic pond water where all defined nitrogen compounds were removed to control for potential background coming from undefined sources of nitrogen, such as yeast extract. Standards were created daily to establish calibration curves ranging from 0.08 mM to 1 mM, which we established as the linear range of the assay with the equipment available. To prepare a sample for NO_2_^−^ analyses, 86.6 µL of Milli-Q water and 40 µL of the Griess reagent were added to target wells in a 96-well plate. 100 µL of the desired subsample was then added to the well and mixed by pipetting up and down prior to incubating at room temperature for 30 min and reading the absorbance on a plate reader at an OD of 584 nm. All samples were blank corrected according to their corresponding medium recipe that included the different terminal electron acceptors added to the medium (Table 1).

Total NH_3_ was measured using a miniaturized version of the indophenol colorimetric method adapted for a 96-well microplate reader (52). Note that in the original method paper, the authors refer to NH_3_ and ammonium (NH_4_^+^) interchangeably to refer to the nitrogen species of interest. In this same paper, the authors do indicate that NH_4_^+^ would dominate speciation in systems with pH <9, such as ours, due to its pKa value of 9.25 (52). In this study, we chose to report the results as NH_3_ because the original citation did not distinguish between the two nitrogen species, and to ensure our reporting is consistent with the literature on AOB, which require NH_3_ as a substrate (53, 54). Standards were prepared in the same background synthetic pond water to control for the pH sensitivity of the colorimetric assay and any undefined sources of nitrogen originating from the yeast extract in the growth medium. Experimental samples needed to undergo a 10-fold dilution in synthetic pond water devoid of defined nitrogen sources to ensure they fell within the linear range of the assay in most cases (Table S1 and Supporting Results). A master mix was created based on the number of tubes (n) that needed to be analyzed, where *n* × 30 µL sodium nitroprusside and *n* × 75 µL of oxidizing solution (hypochlorite) were used. 105 µL of this master mix was aliquoted into 2 mL tubes, followed by the addition of 750 μL of sample or standard. Following this step, 30 µL of phenol solution was added to each tube and gently mixed by pipetting before transferring each sample to a 96-well plate. Plates were incubated in the dark at room temperature for 60 min to allow the color to develop before measuring OD at 640 nm on a plate reader.

### Short-read metagenomic and metatranscriptomic sequencing on enrichment cultures

Suboptimal RNA yields for library preparation were recovered from samples preserved in DNA/RNA shield, and so pellets preserved for DNA extraction were subsequently tested and used for DNA/RNA co-elution for short-read sequencing. Samples were extracted from 2 mL of pelleted cells using the Zymo DNA/RNA Microbiome kit to co-elute each fraction from the same sample. Bead beating homogenization was completed on a VWR Mini beater at 5S speed for 70 s repeated three times in 5-min intervals. DNA samples were purified and concentrated as previously described, but were treated with 4 μL of RNAse A (100 mg/mL) for 10 min at room temperature before purification. RNA was treated with DNase I (Zymo Research) and purified using the RNA Clean and Concentrator Kit-5 (Zymo Research).

DNA inputs of 10 ng were used with NEBNext Ultra II FS with 6–8 cycles of PCR after 15 min of fragmentation and sequenced on a NextSeq 1000 P2 300 cycle cartridge for 150 bp PE reads. RNA libraries were prepared with 100 ng, with Illumina Stranded Total RNA with Ribo-Zero Plus with 15 cycles of PCR on a P2 200 cycle kit for 100 bp PE reads. Initial inoculum samples were first prepared with Ribo-Zero Plus depletion to determine depletion efficiency and design custom depletion probes for the experimental data set. The experimental data set had 1 µL of 50 pmol supplemental probes (IDT) added at the rRNA depletion step (15 µL total volume). Best efforts were attempted in sequencing biological triplicates; however, not all samples extracted produced libraries of sufficient yield and quality for sequencing. Details on RNA probe design to carry out ribosomal RNA depletion are provided in Supplemental methods.

### Bioinformatic analyses

#### PacBio MAGs

Assemblies were performed for each sample’s extraction read set as well as all reads combined using hifiasm_meta v. 0.3-r063.2 (55) and metaMDBG v. 0.3 (56) and subsequently run through the pb-metagenomics HiFi-MAG-Pipeline v. 2.0.2 for binning with completeness-aware binning set to 90% (57). MAGs were all pooled for each sample and assembly type to be dereplicated using dRep v. 3.5.0 (58) with secondary average nucleotide identity (s_ANI) cluster set to 98%.

#### Short-read MAGs

Sequencing reads from the experimental setup were assembled and binned using the nf-core/MAG 2.5.4 pipeline (https://nf-co.re/mag/2.5.4/). Binned MAGs were then assessed separately via checkM2 v. 1.0.1 and filtered to completeness cutoffs of 70% and contamination of 10%. Binned MAGs were combined with dereplicated PacBio MAGs and dereplicated again at 98% ANI for a combined set of 98 MAGs. Final classification was completed with GTDB-TK v. 2.40 using the R220 database. Sylph v. 0.61 (59) was used to classify the reads according to the pooled MAG set used as a reference database across the experimental treatments for comparing sequence abundances.

#### Phylogenetic analyses and virulence factor comparison

Phylogenetic trees were generated using GToTree v. 1.8.4 (60) using taxonomically relevant single-copy gene sets. Reference genomes were downloaded from NCBI to compare against MAGs recovered in this study. Prodigal v. 2.6.3 (61) was used to predict genes on input files, and targets were identified via HMMER3 v. 3.4 (62), aligned with MUSCLE v 5.1 (63), and trimmed with trimAl v. 1.4rev15 (64). Alignments were concatenated for analysis with FastTree2 v. 2.1.11 (65), and taxonomy was determined with TaxonKit v. 0.16.0 (66). Trees were visualized using Dendroscope v. 3.8.10 (67). Abricate v. 1.0.1 (68) was used to identify virulence factors (VFDB, 2025-05-23 [69]) and antibiotic resistance genes (CARD, 2025/05/23, [47]). MAGs were annotated using Bakta v. 1.9.3 with the v. 5.1 database (70). Clinker v. 0.0.31 was used to generate gene cluster comparisons (68).

#### Metabolic annotation

MAG annotation and metabolic summaries were produced using the Distilled Refined Annotation of Metabolism (DRAM) tool v. 1.5.0 (https://github.com/WrightonLabCSU/DRAM/wiki) (71). DRAM outputs three main files that can be used in downstream analyses, including a gene annotation file, a metabolic summary contained in MS Excel, and a tab-delimited file summarizing the percent completion and presence/absence data for essential metabolic pathways. We used custom scripts in R v. 4.3.2 developed using the “tidyverse” package to connect the gene presence/absence data for the nitrogen metabolic module from DRAM to the relative sequence abundance data and taxonomy data (see Data Availability Statement for access to data analyses workflows).

#### Metatranscriptomics analysis

SqueezeMeta v. 1.6.3 (37) processed fastp v. 0.23.4 (72) cleaned DNA and RNA reads from successful libraries prepared from enrichment culture samples using a co-assembly mode on DNA reads with MEGAHIT (73, 74) and a minimum contig length of 500. KEGG annotations were queried using SQMTools v. 1.6.3 (37). Outputs from SqueezeMeta were subsequently processed in R v 4.3.2 to generate DNA and RNA read count figures and conduct data manipulations to identify unique KEGG IDs tied to nitrogen cycling and amino acid modules in KEGG. SqueezeMeta v. 1.7.0b8 was used for RNA read mapping back to the 98 MAGs analyzed in this work.

## Data Availability

Sequencing reads are deposited under BioProject PRJNA1165788. Metagenomic assemblies, annotations, and analysis outputs are available at https://doi.org/10.5281/zenodo.14861210. All R code, supplementary data files, and raw data files with reference to the figures they served as input for in this work have also been provided at https://doi.org/10.5281/zenodo.14861210 and on a dedicated GitHub repository (https://github.com/carleton-envbiotech/Consortium_meta_omics).

## SUPPLEMENTAL MATERIAL

Data Set S1 aem.01984-25-s0001.xlsx Taxonomic and metabolic model summaries for metagenome-assembled genomes (MAGs), relative abundance data, and experimental MAG genome summaries and annotations.

Supplemental text aem.01984-25-s0002.pdf Supplemental methods and results for data analysis.

Supplemental legend aem.01984-25-s0003.docx Legend for Data Set S1.

ASM does not own the copyrights to Supplemental Material that may be linked to, or accessed through, an article. The authors have granted ASM a non-exclusive, world-wide license to publish the Supplemental Material files. Please contact the corresponding author directly for reuse.

## References

[1] Hu J, Yang T, Friman V-P, Kowalchuk GA, Hautier Y, Li M, Wei Z, Xu Y, Shen Q, Jousset A. 2021. Introduction of probiotic bacterial consortia promotes plant growth via impacts on the resident rhizosphere microbiome. Proc Biol Sci 288:20211396. doi:10.1098/rspb.2021.1396 34641724 PMC8511750

[2] Kaya C, Uğurlar F, Ashraf M, Hou D, Kirkham MB, Bolan N. 2024. Microbial consortia-mediated arsenic bioremediation in agricultural soils: current status, challenges, and solutions. Sci Total Environ 917:170297. doi:10.1016/j.scitotenv.2024.170297 38272079

[3] Xu L, Liu B, Huang L, Li Z, Cheng Y, Tian Y, Pan G, Li H, Xu Y, Wu W, Cui Z, Xie L. 2022. Probiotic consortia and their metabolites ameliorate the symptoms of inflammatory bowel diseases in a colitis mouse model. Microbiol Spectr 10:e0065722. doi:10.1128/spectrum.00657-22 35730951 PMC9430814

[4] Molenda O, Quaile AT, Edwards EA. 2016. Dehalogenimonas sp. strain WBC-2 genome and identification of its trans-dichloroethene reductive dehalogenase, TdrA. Appl Environ Microbiol 82:40–50. doi:10.1128/AEM.02017-15 26452554 PMC4702630

[5] Arvanitakis G, Temmerman R, Spök A. 2018. Development and use of microbial-based cleaning products (MBCPs): current issues and knowledge gaps. Food Chem Toxicol 116:3–9. doi:10.1016/j.fct.2017.12.032 29273419

[6] Nunes PSO, Lacerda-Junior GV, Mascarin GM, Guimarães RA, Medeiros FHV, Arthurs S, Bettiol W. 2024. Microbial consortia of biological products: do they have a future? Biol Control 188:105439. doi:10.1016/j.biocontrol.2024.105439

[7] Duhamel M, Edwards EA. 2007. Growth and yields of dechlorinators, acetogens, and methanogens during reductive dechlorination of chlorinated ethenes and dihaloelimination of 1,2-dichloroethane. Environ Sci Technol 41:2303–2310. doi:10.1021/es062010r 17438779

[8] Roell GW, Zha J, Carr RR, Koffas MA, Fong SS, Tang YJ. 2019. Engineering microbial consortia by division of labor. Microb Cell Fact 18:35. doi:10.1186/s12934-019-1083-3 30736778 PMC6368712

[9] Rinke C, Schwientek P, Sczyrba A, Ivanova NN, Anderson IJ, Cheng J-F, Darling A, Malfatti S, Swan BK, Gies EA, Dodsworth JA, Hedlund BP, Tsiamis G, Sievert SM, Liu W-T, Eisen JA, Hallam SJ, Kyrpides NC, Stepanauskas R, Rubin EM, Hugenholtz P, Woyke T. 2013. Insights into the phylogeny and coding potential of microbial dark matter. Nature 499:431–437. doi:10.1038/nature12352 23851394

[10] Rajendhran J, Gunasekaran P. 2011. Microbial phylogeny and diversity: small subunit ribosomal RNA sequence analysis and beyond. Microbiol Res 166:99–110. doi:10.1016/j.micres.2010.02.003 20223646

[11] Cao Z, Yan W, Ding M, Yuan Y. 2022. Construction of microbial consortia for microbial degradation of complex compounds. Front Bioeng Biotechnol 10:1051233. doi:10.3389/fbioe.2022.1051233 36561050 PMC9763274

[12] Rycroft T, Hamilton K, Haas CN, Linkov I. 2019. A quantitative risk assessment method for synthetic biology products in the environment. Sci Total Environ 696:133940. doi:10.1016/j.scitotenv.2019.133940 31446290

[13] Epstein MM, Vermeire T. 2016. Scientific opinion on risk assessment of synthetic biology. Trends Biotechnol 34:601–603. doi:10.1016/j.tibtech.2016.04.013 27234301

[14] Liang Y, Ma A, Zhuang G. 2022. Construction of environmental synthetic microbial consortia: based on engineering and ecological principles. Front Microbiol 13:829717. doi:10.3389/fmicb.2022.829717 35283862 PMC8905317

[15] Gilmore SP, Lankiewicz TS, Wilken SE, Brown JL, Sexton JA, Henske JK, Theodorou MK, Valentine DL, O’Malley MA. 2019. Top-down enrichment guides in formation of synthetic microbial consortia for biomass degradation. ACS Synth Biol 8:2174–2185. doi:10.1021/acssynbio.9b00271 31461261

[16] Environment And Climate Change Canada. 2024. Microbial risk assessment framework (MRAF) under the New Substances Notification Regulations (Organisms) of the Canadian Environmental Protection Act, 1999. Assessments. Available from: https://www.canada.ca/en/environment-climate-change/services/managing-pollution/evaluating-new-substances/biotechnology-living-organisms/microbial-risk-assessment-framework.html. Retrieved 18 Nov 2024.

[17] Jones EJP, Voytek MA, Lorah MM, Kirshtein JD. 2006. Characterization of a microbial consortium capable of rapid and simultaneous dechlorination of 1,1,2,2-tetrachloroethane and chlorinated ethane and ethene intermediates. Bioremediat J 10:153–168. doi:10.1080/10889860601021399

[18] Toth CRA, Luo F, Bawa N, Webb J, Guo S, Dworatzek S, Edwards EA. 2021. Anaerobic benzene biodegradation linked to the growth of highly specific bacterial clades. Environ Sci Technol 55:7970–7980. doi:10.1021/acs.est.1c00508 34041904

[19] La Maestra S, D’Agostini F, Geretto M, Micale RT. 2021. Microbial-based cleaning products as a potential risk to human health: a review. Toxicol Lett 353:60–70. doi:10.1016/j.toxlet.2021.09.013 34626814

[20] Tayabali AF, Coleman G, Crosthwait J, Nguyen KC, Zhang Y, Shwed P. 2017. Composition and pathogenic potential of a microbial bioremediation product used for crude oil degradation. PLoS One 12:e0171911. doi:10.1371/journal.pone.0171911 28178315 PMC5298331

[21] Martínez-Porchas M, Villalpando-Canchola E, Vargas-Albores F. 2016. Significant loss of sensitivity and specificity in the taxonomic classification occurs when short 16S rRNA gene sequences are used. Heliyon 2:e00170. doi:10.1016/j.heliyon.2016.e00170 27699286 PMC5037269

[22] Callahan BJ, Wong J, Heiner C, Oh S, Theriot CM, Gulati AS, McGill SK, Dougherty MK. 2019. High-throughput amplicon sequencing of the full-length 16S rRNA gene with single-nucleotide resolution. Nucleic Acids Res 47:e103. doi:10.1093/nar/gkz569 31269198 PMC6765137

[23] Kim C, Pongpanich M, Porntaveetus T. 2024. Unraveling metagenomics through long-read sequencing: a comprehensive review. J Transl Med 22:111. doi:10.1186/s12967-024-04917-1 38282030 PMC10823668

[24] Eisenhofer R, Nesme J, Santos-Bay L, Koziol A, Sørensen SJ, Alberdi A, Aizpurua O. 2024. A comparison of short-read, HiFi long-read, and hybrid strategies for genome-resolved metagenomics. Microbiol Spectr 12:e0359023. doi:10.1128/spectrum.03590-23 38451230 PMC10986573

[25] Hsieh YE, Tandon K, Verbruggen H, Nikoloski Z. 2025. Integration of metatranscriptomics data improves the predictive capacity of microbial community metabolic models. ISME J 19:wraf109. doi:10.1093/ismejo/wraf109 40448581 PMC12203112

[26] Buetas E, Jordán-López M, López-Roldán A, D’Auria G, Martínez-Priego L, De Marco G, Carda-Diéguez M, Mira A. 2024. Full-length 16S rRNA gene sequencing by PacBio improves taxonomic resolution in human microbiome samples. BMC Genomics 25:310. doi:10.1186/s12864-024-10213-5 38528457 PMC10964587

[27] Freeman CN, Russell JN, Yost CK. 2024. Temporal metagenomic characterization of microbial community structure and nitrogen modification genes within an activated sludge bioreactor system. Microbiol Spectr 12:e0283223. doi:10.1128/spectrum.02832-23 38018980 PMC10783093

[28] Johnston J, LaPara T, Behrens S. 2019. Composition and dynamics of the activated sludge microbiome during seasonal nitrification failure. Sci Rep 9:4565. doi:10.1038/s41598-019-40872-4 30872659 PMC6418219

[29] Cai M, Ng S-K, Lim CK, Lu H, Jia Y, Lee PKH. 2018. Physiological and metagenomic characterizations of the synergistic relationships between ammonia- and nitrite-oxidizing bacteria in freshwater nitrification. Front Microbiol 9:280. doi:10.3389/fmicb.2018.00280 29535685 PMC5835065

[30] Norton JM, Klotz MG, Stein LY, Arp DJ, Bottomley PJ, Chain PSG, Hauser LJ, Land ML, Larimer FW, Shin MW, Starkenburg SR. 2008. Complete genome sequence of Nitrosospira multiformis, an ammonia-oxidizing bacterium from the soil environment. Appl Environ Microbiol 74:3559–3572. doi:10.1128/AEM.02722-07 18390676 PMC2423025

[31] Grundmann GL, Dechesne A, Bartoli F, Flandrois JP, Chassé JL, Kizungu R. 2001. Spatial modeling of nitrifier microhabitats in soil. Soil Science Soc of Amer J 65:1709–1716. doi:10.2136/sssaj2001.1709

[32] Ibarbalz FM, Orellana E, Figuerola ELM, Erijman L. 2016. Shotgun metagenomic profiles have a high capacity to discriminate samples of activated sludge according to wastewater type. Appl Environ Microbiol 82:5186–5196. doi:10.1128/AEM.00916-16 27316957 PMC4988215

[33] Zhang Y, Deng Y, Wang C, Li S, Lau FTK, Zhou J, Zhang T. 2024. Effects of operational parameters on bacterial communities in Hong Kong and global wastewater treatment plants. mSystems 9:e0133323. doi:10.1128/msystems.01333-23 38411061 PMC10949511

[34] Siripong S, Rittmann BE. 2007. Diversity study of nitrifying bacteria in full-scale municipal wastewater treatment plants. Water Res 41:1110–1120. doi:10.1016/j.watres.2006.11.050 17254627

[35] Wu R-N, Meng H, Wang Y-F, Lan W, Gu J-D. 2017. A more comprehensive community of ammonia-oxidizing archaea (AOA) revealed by genomic DNA and RNA analyses of amoA gene in subtropical acidic forest soils. Microb Ecol 74:910–922. doi:10.1007/s00248-017-1045-4 28808742

[36] Shakya M, Lo C-C, Chain PSG. 2019. Advances and challenges in metatranscriptomic analysis. Front Genet 10:904. doi:10.3389/fgene.2019.00904 31608125 PMC6774269

[37] Tamames J, Puente-Sánchez F. 2018. SqueezeMeta, a highly portable, fully automatic metagenomic analysis pipeline. Front Microbiol 9:3349. doi:10.3389/fmicb.2018.03349 30733714 PMC6353838

[38] Sayavedra-Soto LA, Hommes NG, Alzerreca JJ, Arp DJ, Norton JM, Klotz MG. 1998. Transcription of the amoC, amoA and amoB genes in Nitrosomonas europaea and Nitrosospira sp. NpAV. FEMS Microbiol Lett 167:81–88. doi:10.1111/j.1574-6968.1998.tb13211.x 9785456

[39] Berube PM, Samudrala R, Stahl DA. 2007. Transcription of all amoC copies is associated with recovery of Nitrosomonas europaea from ammonia starvation. J Bacteriol 189:3935–3944. doi:10.1128/JB.01861-06 17384196 PMC1913382

[40] Phan HV, Yasuda S, Oba K, Tsukamoto H, Hori T, Kuroiwa M, Terada A. 2025. Active bacteria driving N_2_O mitigation and dissimilatory nitrate reduction to ammonium in ammonia recovery bioreactors. ISME J 19:wraf021. doi:10.1093/ismejo/wraf021 39913347 PMC11879220

[41] Yuan H, Jia B, Zeng Q, Zhou Y, Wu J, Wang H, Fang H, Cai Y, Li Q. 2022. Dissimilatory nitrate reduction to ammonium (DNRA) potentially facilitates the accumulation of phosphorus in lake water from sediment. Chemosphere 303:134664. doi:10.1016/j.chemosphere.2022.134664 35460675

[42] Proust L, Sourabié A, Pedersen M, Besançon I, Haudebourg E, Monnet V, Juillard V. 2019. Insights into the complexity of yeast extract peptides and their utilization by Streptococcus thermophilus. Front Microbiol 10:906. doi:10.3389/fmicb.2019.00906 31133999 PMC6524704

[43] Cyprowski M, Stobnicka-Kupiec A, Ławniczek-Wałczyk A, Bakal-Kijek A, Gołofit-Szymczak M, Górny RL. 2018. Anaerobic bacteria in wastewater treatment plant. Int Arch Occup Environ Health 91:571–579. doi:10.1007/s00420-018-1307-6 29594341 PMC6002452

[44] Bolufer Cruañes C, Ouradou A, Pineault S, Boivin M-C, Huot C, Bédard E. 2024. Uncovering wastewater treatment plants as possible sources of legionellosis clusters through spatial statistics approach and environmental analysis. Environ Sci Pollut Res 31:45234–45245. doi:10.1007/s11356-024-34019-w 38961023

[45] van den Berg H, Lodder W, Bartels A, Brandsema P, Vermeulen L, Lynch G, Euser S, de Roda Husman AM. 2023. Legionella detection in wastewater treatment plants with increased risk for Legionella growth and emission. J Water Health 21:1291–1302. doi:10.2166/wh.2023.164 37756196

[46] Gomez-Valero L, Chiner-Oms A, Comas I, Buchrieser C. 2019. Evolutionary dissection of the Dot/Icm system based on comparative genomics of 58 Legionella species. Genome Biol Evol 11:2619–2632. doi:10.1093/gbe/evz186 31504472 PMC6761968

[47] Alcock BP, Huynh W, Chalil R, Smith KW, Raphenya AR, Wlodarski MA, Edalatmand A, Petkau A, Syed SA, Tsang KK, et al.. 2023. CARD 2023: expanded curation, support for machine learning, and resistome prediction at the Comprehensive Antibiotic Resistance Database. Nucleic Acids Res 51:D690–D699. doi:10.1093/nar/gkac920 36263822 PMC9825576

[48] Papalia M, González-Espinosa F, Castedo FQ, Gutkind G, Ramírez MS, Power P, Radice M. 2024. Genetic and biochemical characterization of AXC-2 from Achromobacter ruhlandii. Pathogens 13:115. doi:10.3390/pathogens13020115 38392853 PMC10893412

[49] Hong SK, Sung JY, Lee HJ, Oh M-D, Park SS, Kim E-C. 2013. First case of Mycobacterium longobardum infection. Ann Lab Med 33:356–359. doi:10.3343/alm.2013.33.5.356 24003427 PMC3756241

[50] Lekic N, Rosenberg AE, Askari M. 2018. Mycobacterium longobardum infection in the hand. J Hand Surg Am 43:491. doi:10.1016/j.jhsa.2017.09.008 29032284

[51] Shachor-Meyouhas Y, Geffen Y, Arad-Cohen N, Zaidman I, Ben-Barak A, Davidson S, Kassis I. 2014. Mycobacterium phocaicum bacteremia: an emerging infection in pediatric hematology-oncology patients. Pediatr Infect Dis J 33:1299–1301. doi:10.1097/INF.0000000000000477 25037036

[52] Tzollas NM, Zachariadis GA, Anthemidis AN, Stratis JA. 2010. A new approach to indophenol blue method for determination of ammonium in geothermal waters with high mineral content. Int J Environ Anal Chem 90:115–126. doi:10.1080/03067310902962528

[53] Soliman M, Eldyasti A. 2018. Ammonia-Oxidizing Bacteria (AOB): opportunities and applications—a review. Rev Environ Sci Biotechnol 17:285–321. doi:10.1007/s11157-018-9463-4

[54] Suzuki I, Dular U, Kwok SC. 1974. Ammonia or ammonium ion as substrate for oxidation by Nitrosomonas europaea cells and extracts. J Bacteriol 120:556–558. doi:10.1128/jb.120.1.556-558.1974 4422399 PMC245802

[55] Feng X, Cheng H, Portik D, Li H. 2022. Metagenome assembly of high-fidelity long reads with hifiasm-meta. Nat Methods 19:671–674. doi:10.1038/s41592-022-01478-3 35534630 PMC9343089

[56] Benoit G, Raguideau S, James R, Phillippy AM, Chikhi R, Quince C. 2024. High-quality metagenome assembly from long accurate reads with metaMDBG. Nat Biotechnol 42:1378–1383. doi:10.1038/s41587-023-01983-6 38168989 PMC11392814

[57] Portik DM, Brown CT, Pierce-Ward NT. 2022. Evaluation of taxonomic classification and profiling methods for long-read shotgun metagenomic sequencing datasets. BMC Bioinformatics 23:541. doi:10.1186/s12859-022-05103-0 36513983 PMC9749362

[58] Olm MR, Brown CT, Brooks B, Banfield JF. 2017. dRep: a tool for fast and accurate genomic comparisons that enables improved genome recovery from metagenomes through de-replication. ISME J 11:2864–2868. doi:10.1038/ismej.2017.126 28742071 PMC5702732

[59] Shaw J, Yu YW. 2025. Rapid species-level metagenome profiling and containment estimation with sylph. Nat Biotechnol 43:1348–1359. doi:10.1038/s41587-024-02412-y 39379646 PMC12339375

[60] Lee MD. 2019. GToTree: a user-friendly workflow for phylogenomics. Bioinformatics 35:4162–4164. doi:10.1093/bioinformatics/btz188 30865266 PMC6792077

[61] Hyatt D, Chen G-L, Locascio PF, Land ML, Larimer FW, Hauser LJ. 2010. Prodigal: prokaryotic gene recognition and translation initiation site identification. BMC Bioinformatics 11:119. doi:10.1186/1471-2105-11-119 20211023 PMC2848648

[62] Eddy SR. 2011. Accelerated profile HMM searches. PLoS Comput Biol 7:e1002195. doi:10.1371/journal.pcbi.1002195 22039361 PMC3197634

[63] Edgar RC. 2022. Muscle5: High-accuracy alignment ensembles enable unbiased assessments of sequence homology and phylogeny. Nat Commun 13:6968. doi:10.1038/s41467-022-34630-w 36379955 PMC9664440

[64] Capella-Gutiérrez S, Silla-Martínez JM, Gabaldón T. 2009. trimAl: a tool for automated alignment trimming in large-scale phylogenetic analyses. Bioinformatics 25:1972–1973. doi:10.1093/bioinformatics/btp348 19505945 PMC2712344

[65] Price MN, Dehal PS, Arkin AP. 2025. FastTree 2 – approximately maximum-likelihood trees for large alignments. PLoS One 5:e9490. doi:10.1371/journal.pone.0009490 PMC2835736 20224823

[66] Shen W, Ren H. 2021. TaxonKit: a practical and efficient NCBI taxonomy toolkit. J Genet Genomics 48:844–850. doi:10.1016/j.jgg.2021.03.006 34001434

[67] Huson DH, Scornavacca C. 2012. Dendroscope 3: an interactive tool for rooted phylogenetic trees and networks. Syst Biol 61:1061–1067. doi:10.1093/sysbio/sys062 22780991

[68] Seemann T. 2025. Tseemann/abricate. Perl. https://github.com/tseemann/abricate.

[69] Chen L, Zheng D, Liu B, Yang J, Jin Q. 2016. VFDB 2016: hierarchical and refined dataset for big data analysis—10 years on. Nucleic Acids Res 44:D694–D697. doi:10.1093/nar/gkv1239 26578559 PMC4702877

[70] Schwengers O, Jelonek L, Dieckmann MA, Beyvers S, Blom J, Goesmann A. 2021. Bakta: rapid and standardized annotation of bacterial genomes via alignment-free sequence identification. Microb Genom 7. doi:10.1099/mgen.0.000685 PMC8743544 34739369

[71] Shaffer M, Borton MA, McGivern BB, Zayed AA, La Rosa SL, Solden LM, Liu P, Narrowe AB, Rodríguez-Ramos J, Bolduc B, Gazitúa MC, Daly RA, Smith GJ, Vik DR, Pope PB, Sullivan MB, Roux S, Wrighton KC. 2020. DRAM for distilling microbial metabolism to automate the curation of microbiome function. Nucleic Acids Res 48:8883–8900. doi:10.1093/nar/gkaa621 32766782 PMC7498326

[72] Chen S, Zhou Y, Chen Y, Gu J. 2018. fastp: an ultra-fast all-in-one FASTQ preprocessor. Bioinformatics 34:i884–i890. doi:10.1093/bioinformatics/bty560 30423086 PMC6129281

[73] Li D, Liu C-M, Luo R, Sadakane K, Lam T-W. 2015. MEGAHIT: an ultra-fast single-node solution for large and complex metagenomics assembly via succinct de Bruijn graph. Bioinformatics 31:1674–1676. doi:10.1093/bioinformatics/btv033 25609793

[74] Li D, Luo R, Liu C-M, Leung C-M, Ting H-F, Sadakane K, Yamashita H, Lam T-W. 2016. MEGAHIT v1.0: a fast and scalable metagenome assembler driven by advanced methodologies and community practices. Methods 102:3–11. doi:10.1016/j.ymeth.2016.02.020 27012178

