# Supplementary material for: Multi-omics provides functional insights and underscores practical challenges in assessing the composition and performance of a nitrifying microbial consortium
Source: Appl Environ Microbiol. 2025 Dec 29;92(1):e01984-25. doi: 10.1128/aem.01984-25 (PMC12838200; doi:10.1128/aem.01984-25)
Supplement: Supplemental text — Supplemental methods and results for data analysis. [file aem.01984-25-s0002.pdf]

Supporting information: Multi-omics provides functional insights and underscores practical challenges in assessing the composition and performance of a nitrifying microbial consortium

Supporting Methods

rRNA probe design

Supporting Results

Nitrogen chemistry analyses

Supporting data, figures and tables

Supplementary Data 1: Taxonomic and metabolic model summaries for metagenome-assembled genomes (MAGs), relative abundance data, and experimental MAG genome summaries and annotations

- Sheet 1: Summary information from the long-read PacBio sequencing carried out on starting consortium material
- Sheet 2: DRAM metabolism summary output for energy conserving pathways
- Sheet 3: Raw O.D. 600 nm, pH, and nitrogen chemistry data
- Sheet 4: Summary information from 98 MAGs recovered from experimental conditions
- Sheet 5: Abricate results from 98 MAGs queried against the VFDB
- Sheet 6: Abricate results from 98 MAGs queried against CARD

Figure S1: Cell growth based on O.D. 600 nm and pH data for enrichment cultures

Figure S2: NMDS analyses at the MAG level omitting the aerobic cultures

Figure S3: % RNA reads mapped to key genes involved in nitrogen redox cycling

Figure S4: % RNA reads mapped to metagenome-assembled genomes (MAGs)

Figure S5: Taxonomic classification of % RNA mapped to nitrogen cycling genes

Figure S6: Read counts based on RNA sequencing for genes involved in amino acid metabolism

Figure S7: Phylogenetics of an *Achromobacter denitrificans* MAG recovered from the consortium.

Figure S8: Phylogenetics of the *Mycobacteriaceae* MAGs recovered from the consortium.

Table S1: Calibration curve data from colorimetric ammonia detection method

Supporting Methods

rRNA Probe design

The depletion of ribosomal RNA (rRNA) can be a significant challenge when working with complex mixtures of organisms, particularly those with genes divergent from the standard approaches designed for commonly used models in laboratory settings. This depletion is necessary to reduce sequence efforts required to sample transcripts from the overall community as rRNA can make up between 90-99% of the total rRNA and a lack of polyadenylation within prokaryotes makes laboratory approaches non-trivial (1–3). The description of an approach previously applied to complex microbial communities was adapted into a modified workflow described below (4).

Custom depletion probes were first designed using reads mapping to ribosomal RNA with sortmerna v 4.3 using the default database after depletion by Illumina's RiboZero Plus. These reads were mapped using bowtie2 v. 2.5.2 against rRNAs identified with barrnap v 0.9 from a subset of assembled MAGs (combined read sets for each sample using the metaMDBG assembly bins only) and duplicate calls were removed. Reads were then mapped against these targets with the unmapped portions assembled using rnaSPAdes v 3.13.0 to be annotated with barrnap again, duplicates removed, and added to the unique rRNAs from the MAG assemblies. Reads were then mapped back to these rRNAs and top regions were identified with bedtools and clustered via CD-Hit v 4.8.1 at multiple identities to minimize the number of rRNA sequences used for probe design. Custom probes were then designed against representative non-depleted rRNA targets as determined by Illumina's internal pipeline to generate 50mer oligos purchased from IDT, which has a limitation of a total of 384 probes at 50 pmol concentration. This limitation necessitated ordering two sets of probes, Set A with 285 probes and Set B with 238. Each pool contained a subset of the top 25 rRNA sequences identified clustered at 80% identity. These pools were combined to 50 pmol total concentration. Depletion efficiency was determined via sortmerna v. 4.3 as above. Following depletion with Illumina Ribo-Zero Plus, two replicates of the initial inoculum had an average of 73.5% of reads mapping to rRNA. After adding custom probes designed against the rRNA remaining after Ribo-Zero Plus depletion, our metatranscriptomic samples ranged from 74.8% to 2.10% rRNA, with the average being 35.9%.

Supporting Results

Nitrogen chemical analyses

There are some analytical inconsistencies related to NH_3 in this work that warrant further explanation. Our initial measurements for NH_3 at time 0 led to an overestimation based on the theoretical nitrogen budget of the medium. The total nitrogen budget calculated from defined nitrogen sources in the synthetic pond water medium is 3.11 mM (*i.e.,* 0.84 mM of KNO_3, 0.87 mM of $NaNO_2$, and 1.4 mM of NH_4Cl). The iron reduction metabolic treatment has an additional of 1 mM Fe^{III}-NH_4^+-citrate that provides an additional 1 mM of nitrogen. At most, we would expect 4.11 mM of nitrogen in the medium and our NH_3 measurements at time 0 exceeded that even in sterile controls (see **Figure 2**).

Although it is possible that the 1 g L^{-1} of yeast extract in the medium released NH_3 given that this reagent can contain ~10 % total nitrogen (equivalent to an additional 3.57 mM total nitrogen in the medium)(per Certificate of Analysis, Sigma-Aldrich Product Number Y0875, ThermoFisher product H26769.36), we anticipated this and prepared all calibration curves for colorimetric assays in synthetic pond water containing yeast extract but devoid of additional defined nitrogen sources to control for potential background effects (see **Methods**). Furthermore, we ensured all calibration curves were conducted at the same pH as the starting growth medium to avoid shifts in NH_3 vs NH_4^+ speciation due to pH. We can further rule out these uncertainties stem from NH_3/NH_4^+ buffering because abiotic controls would have had sufficient time to equilibrate once the medium was added to bottles and prior to subsampling (*i.e.,* > 48 hours in the anaerobic chamber). It is possible that the carry-over of biomass in the biotic treatments contributed to this fluctuation, but it seems unlikely as we would have expected more of a difference between bottles that received live cells vs sterile controls. By the same logic, we can rule out rapid amino acid metabolism contributing to these discrepancies in live cultures because we would have expected a larger difference between treatments with and without cells.

Based on preliminary optimization efforts, we analyzed all samples for NH_3 undiluted alongside 10-fold dilutions to capture a wide range of NH_3 concentrations that fell below the upper limit of detection of this assay (*i.e.,* 3 mM). Outside of the initial time point, the undiluted samples routinely overloaded the absorbance detection on the microplate reader used in this work providing semi-quantitative evidence of high NH_3 production. As such, we chose to present the 10-fold dilution data that fell within the linear range of the NH_3 assay for all samples to be consistent. We cannot discount that small analytical variances may have led to overestimations for the time 0 point after applying the 10X dilution factor. For all treatments except the iron reducing one, which contained additional nitrogen, the values reported for the undiluted samples at time 0 are more in line with the theoretical addition of NH_4Cl to the medium (ca. 1.36 mM to 2.15 mM vs the target of 1.4 mM, see sheet #3 in **Supplementary Data 1**). The calibration curve tied to the start of the experiment also had the lowest slope coefficient of all the assays we ran (see **Table S1**). When applied an average of all slopes associated with NH_3 in this work to the 10-fold diluted data the values were consistently lower, albeit not totally in line with the target.

We suspect multiple variables contributed to the analytical variance observed at time 0. Given that the undiluted NH_3 samples at time 0 align with the theoretical starting composition of the medium and that subsequent measurements in abiotic controls using 10-fold diluted samples also aligned with the theoretical NH_3 in the medium, we think it is appropriate to carry out a temporal comparison of NH_3 concentrations but using the 10-fold diluted data for consistency.

Note that one of our goals in this study was to apply a low cost and high throughput method to identify cultures with nitrifying phenotypes to focus on more detailed analyses of sequencing data. We have successfully carried out that phenotypic characterization which is further supported by independent analyses of the oxidized byproducts NO_2^- and NO_3^- where undiluted and 10-fold diluted samples provided concentrations within the expected ranges of the synthetic pondwater's composition, respectively.

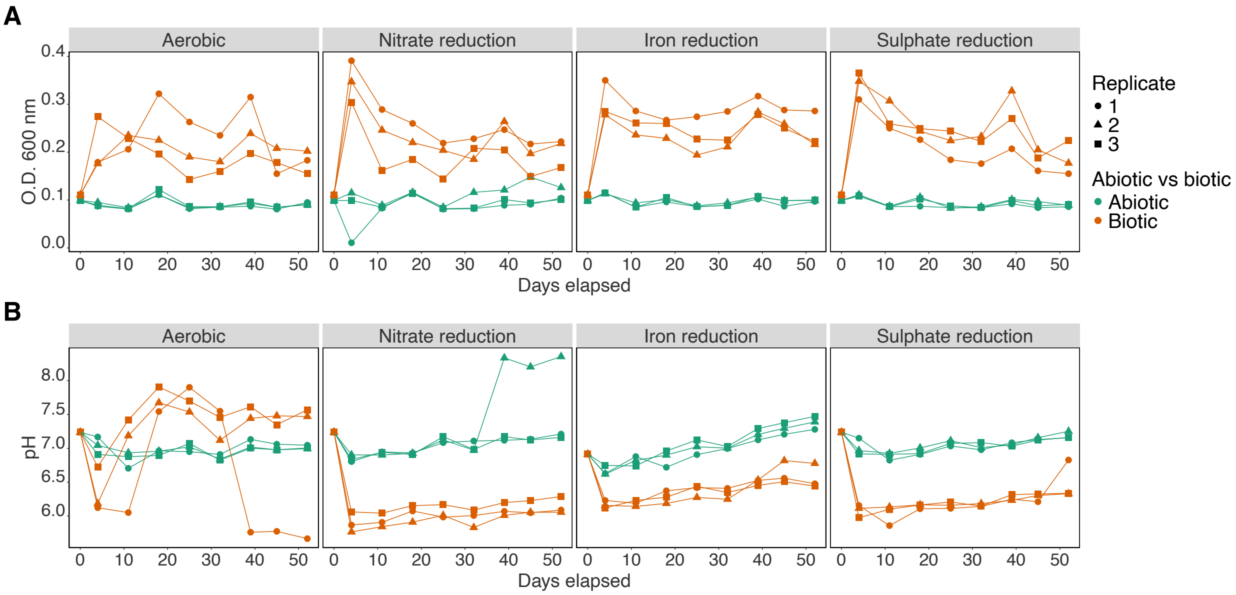

Figure S1: Cell growth measured by optical density at 600 nm (O.D. 600 nm) and pH measurements for enrichment cultures inoculated with starting material from the ammonia oxidizing consortium in development and grown along a redox gradient in synthetic pond water. Bottles that received live cells are labeled as biotic whereas sterile bottles are labelled as abiotic with both being colour-coded. Individual replicates are represented by different shapes. Bottles from the aerobic metabolic treatment had their headspace renewed daily with sterile room air during working days (*i.e.,* Monday to Friday) whereas all anaerobic treatments were provided with 97% N_2/3% H_2 as headspace and handled in an anaerobic glovebox. The nitrate reduction treatment was grown with the NO_3^- already present in the medium acting as an electron acceptor whereas the iron reduction and sulphate reduction treatments were amended with Fe^{III} and SO_4^{2-} as terminal electron acceptors, respectively. Abiotic vs biotic treatments are colour coded and triplicates are denoted by different shapes.

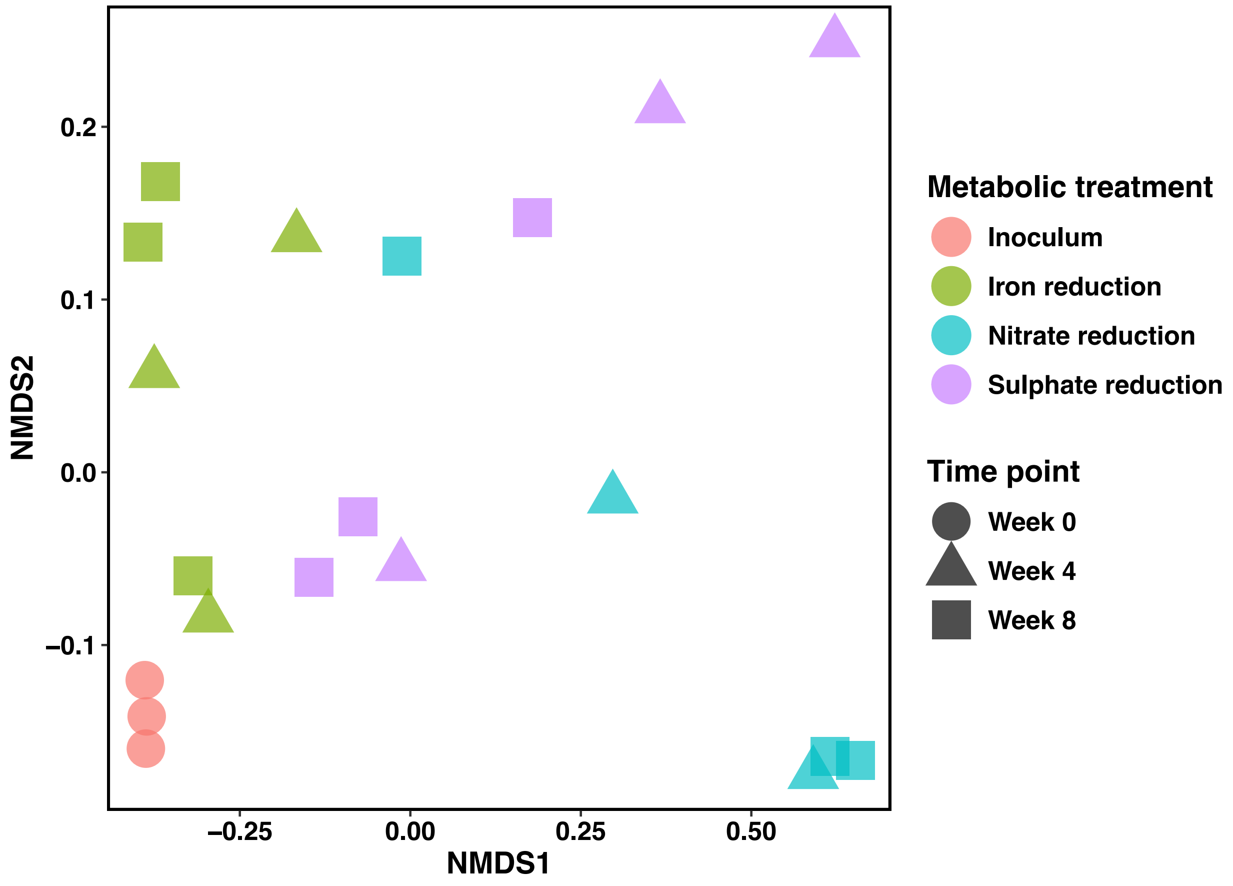

Figure S2: Non-metric multidimensional scaling (NMDS) based on the relative abundance of 98 metagenome-assembled-genomes recovered from enrichment cultures grown along a redox gradient for eight weeks. Samples obtained from cultures subject to the aerobic treatment were omitted from NMDS analyses. The Bray-Curtis dissimilarity index was used to generate a distance matrix required for NMDS analysis. The default commands 'metaMDS' from the 'vegan' package was used to run 20 iterations of the NMDS ordination, which provided a stress value of 0.0389. Metabolic treatments that capture the redox gradient have been colour-coded and the different times points used to subsample biomass for DNA sequencing have been identified by different shapes.

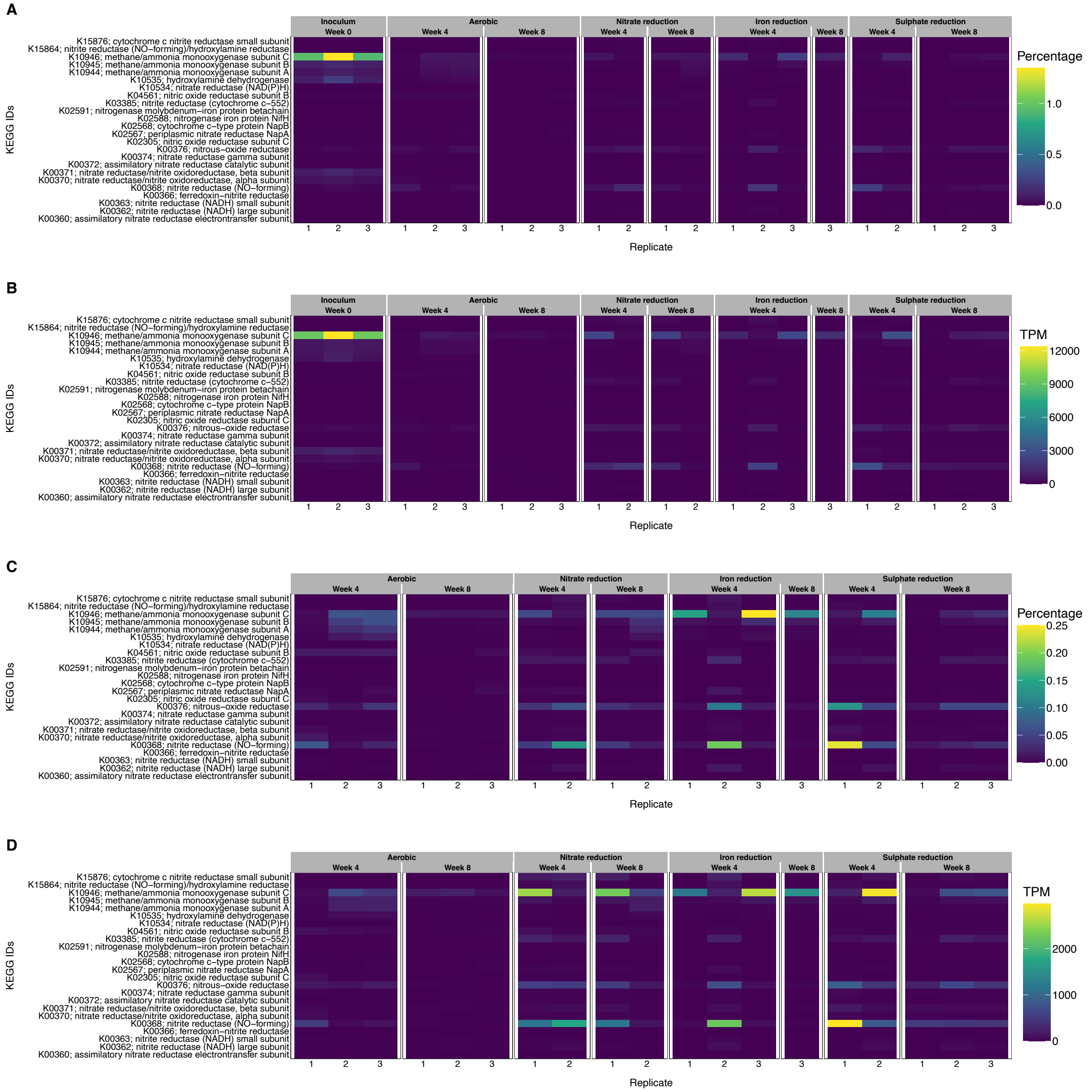

Figure S3: Gene counts based on RNA read mapping to the coassembly data for key genes involved in nitrification and denitrification. Panel A) shows all the % RNA data acquired for pathways of interest. Panel C) shows the same data that has been rescaled after removing extreme values for KEGG 10946 coding AmoC subunit. Panel B) and D) show the same data in TPM. RNA read mapping was performed in SqueezeMeta and data were subsequently exported using the plotFunctions function in SQMtools (v 1.6.3) and R v 4.3.2 to refine the visualizations.

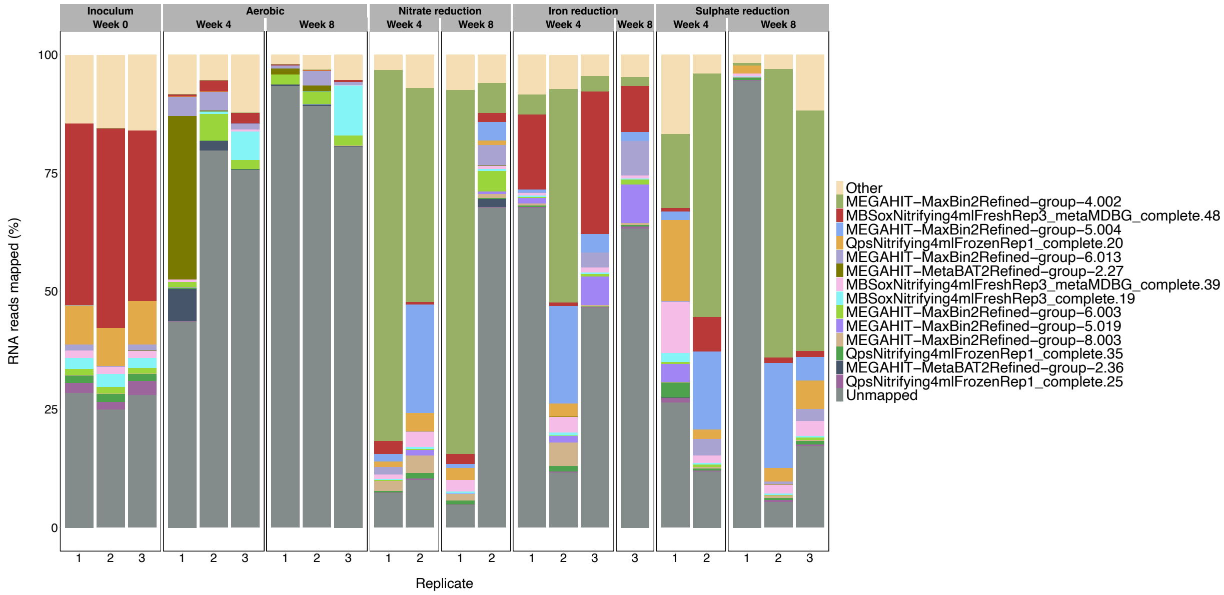

Figure S4: Percent of RNA reads mapped to metagenome-assembled-genomes (genome bins) obtained from enrichment cultures grown along a redox gradient for eight weeks using the nitrifying consortium in development as inoculum material. RNA read mapping was performed in SqueezeMeta and data were subsequently exported using the plotBins function in SQMtools (v 1.6.3) and R v 4.3.2 to refine the visualizations.

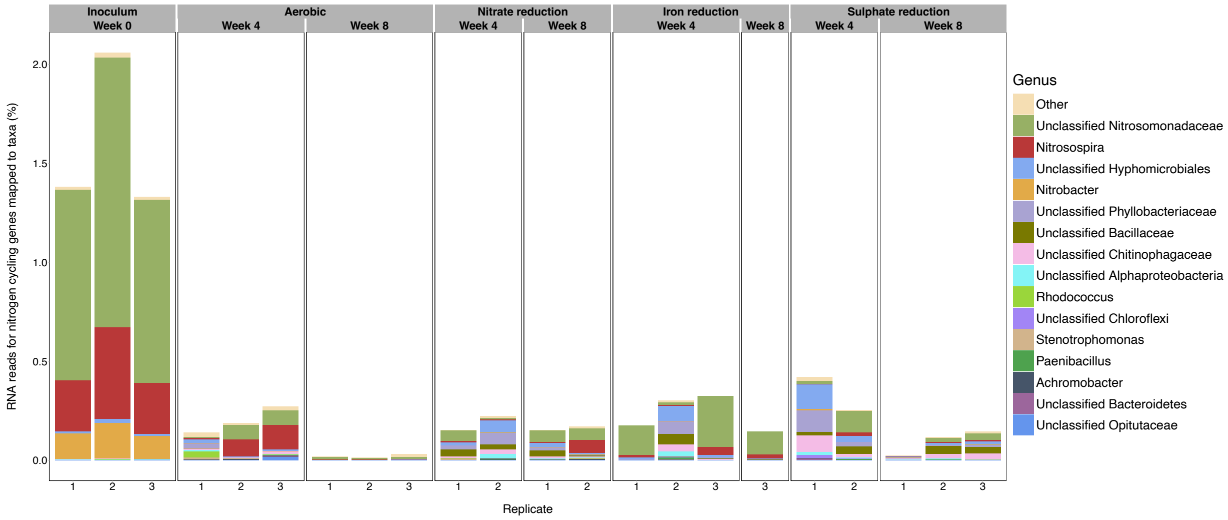

Figure S5: Percent RNA reads mapped and taxonomically-classified to nitrogen cycling genes detected in metagenome-assembled-genomes (genome bins) obtained from enrichment cultures grown along a redox gradient for eight weeks using the nitrifying consortium in development as inoculum materials. RNA read mapping was performed in SqueezeMeta and data were subsequently exported using the plotTaxonomy function in SQMtools (v 1.6.3) and R v 4.3.2 to refine the visualizations.

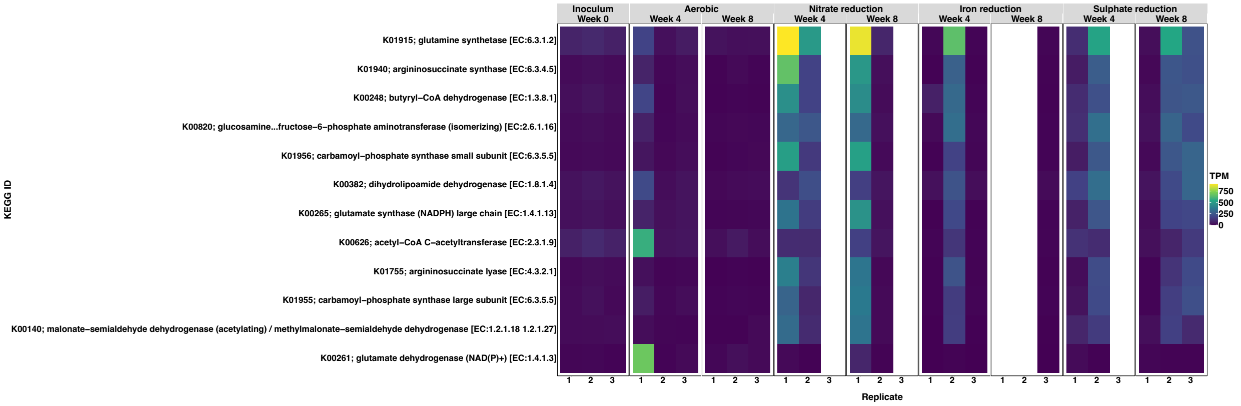

Figure S6: Top 25 RNA read counts for key genes involved in amino acid metabolism from metagenomes for the starting consortium material (week 0) and samples taken from enrichment cultures grown along a redox gradient (Weeks 4 and 8). Amino acid pathways were subset using the subset function for KEGG modules in SqueezeMeta and data were exported in tab-delimited format to refine data visualizations in R v 4.3.2.

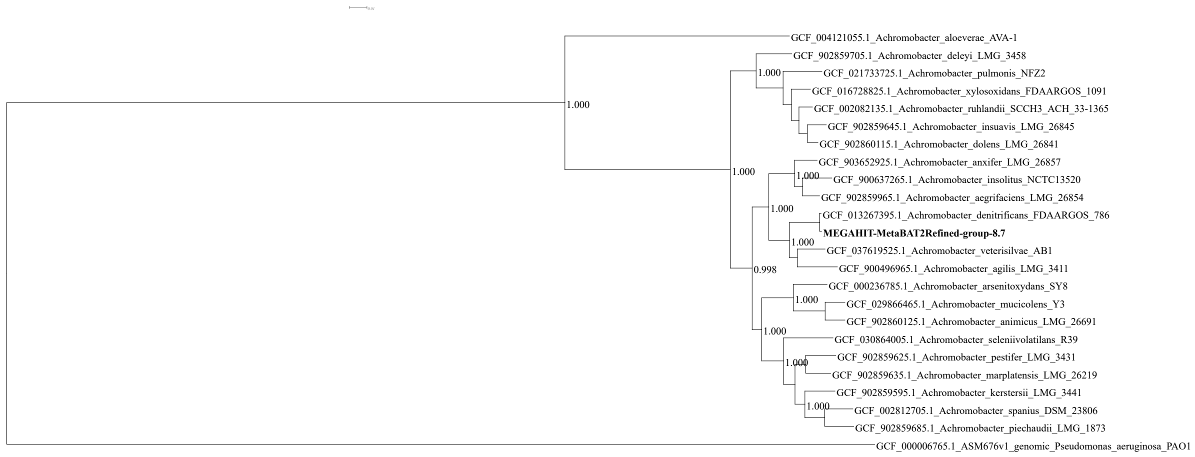

Figure S7: Phylogenetics of an *Achromobacter denitrificans* MAG recovered from the consortium experiment. A phylogenetic tree was generated from core genes identified via GtoTree using the Gammaproteobaceria HMM profile (up to 172 genes) and was rooted on *Pseudomonas aeruginosa* PAO1.

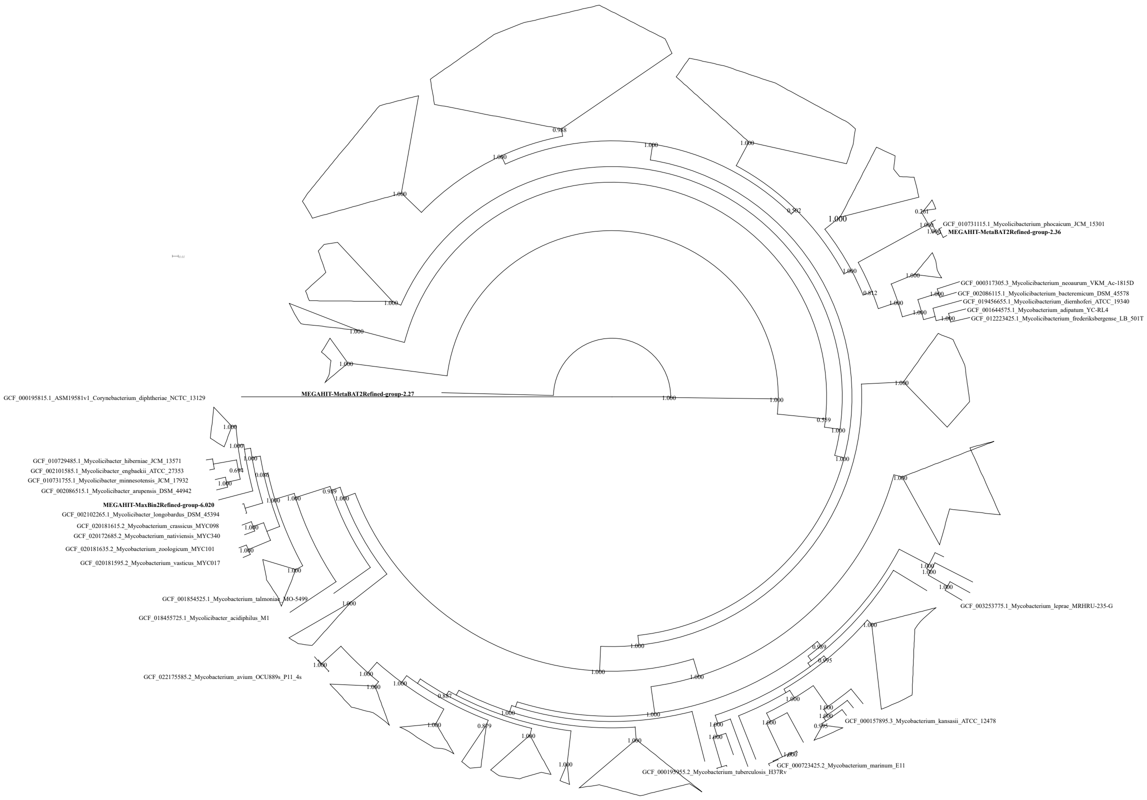

Figure S8: Phylogenetics of the *Mycobacteriaceae* MAGs recovered from the consortium experiment. A phylogenetic tree was generated from core genes identified via GtoTree using the Actinobacteria HMM (up to 138 genes) and was rooted on *Corynebacterium diphtheriae* NCTC 13129. Clades were collapsed and nodes were hidden for clarity and to highlight relationships of reference genomes of species of interest.

Table S1: Calibration curve slope equations (y = mx + b) for the NH_3 colorimetric assay.

Date	Time point	Slope coefficient	Intercept	R^2
2023-06-19	Week 0	1.3023x	+0.4819	0.9960
2023-06-23	Week 1	2.5309x	+0.173	0.9894
2023-06-30	Week 2	1.4854x	-0.0122	0.9793
2023-07-07	Week 3	2.1635x	-0.0544	0.9696
2023-07-14	Week 4	2.9652x	-0.1928	0.9981
2023-07-21	Week 5	2.9307x	-0.1457	0.9993
2023-07-28	Week 6	2.9195x	+0.0336	0.9952
2023-08-03	Week 7	3.0905x	-0.3718	0.9517
2023-08-10	Week 8	1.9216x	-0.1274	0.9598

Supporting References

1. Giannoukos G, Ciulla DM, Huang K, Haas BJ, Izard J, Levin JZ, Livny J, Earl AM, Gevers D, Ward DV, Nusbaum C, Birren BW, Gnirke A. 2012. Efficient and robust RNA-seq process for cultured bacteria and complex community transcriptomes. Genome Biol 13:r23.

2. Wangsanuwat C, Heom KA, Liu E, O’Malley MA, Dey SS. 2020. Efficient and cost-effective bacterial mRNA sequencing from low input samples through ribosomal RNA depletion. BMC Genomics 21:717.

3. Palazzo AF, Lee ES. 2015. Non-coding RNA: what is functional and what is junk? Front Genet 6:2.

4. Rational probe design for efficient rRNA depletion and improved metatranscriptomic analysis of human microbiomes | BMC Microbiology | Full Text. https://bmcmicrobiol.biomedcentral.com/articles/10.1186/s12866-023-03037-y. Retrieved 9 July 2025.
