# Supplementary material for: Multi-omics provides functional insights and underscores practical challenges in assessing the composition and performance of a nitrifying microbial consortium
Source: Appl Environ Microbiol. 2025 Dec 29;92(1):e01984-25. doi: 10.1128/aem.01984-25 (PMC12838200; doi:10.1128/aem.01984-25)
Supplement: Supplemental legend — Legend for Data Set S1. [file aem.01984-25-s0003.docx]

**Supplementary Data Legend**

**Supplementary Data 1:** Taxonomic and metabolic model summaries for metagenome-assembled genomes (MAGs), relative abundance data, and experimental MAG genome summaries and annotations

- Sheet 1: Summary information from the long-read PacBio sequencing carried out on starting consortium material
- Sheet 2: DRAM metabolism summary output for energy conserving pathways
- Sheet 3: Raw O.D. 600 nm, pH, and nitrogen chemistry data
- Sheet 4: Summary information from 98 MAGs recovered from experimental conditions
- Sheet 5: Abricate results from 98 MAGs queried against the VFDB
- Sheet 6: Abricate results from 98 MAGs queried against CARD
